# The Effects of One Anastomosis Gastric Bypass Surgery on the Gastrointestinal Tract

**DOI:** 10.3390/nu14020304

**Published:** 2022-01-12

**Authors:** Osnat Kaniel, Shiri Sherf-Dagan, Amir Szold, Peter Langer, Boris Khalfin, Yafit Kessler, Asnat Raziel, Nasser Sakran, Yair Motro, David Goitein, Jacob Moran-Gilad

**Affiliations:** 1 Department of Health Policy and Management, Guilford Glazer Faculty of Business and Management, Ben-Gurion University of the Negev, Beer-Sheva 84105, Israel; osnatyy@gmail.com; 2 Department of Nutritional Sciences, School of Health Sciences, Ariel University, Ariel 40700, Israel; shirisherf@gmail.com (S.S.-D.); yafitke@gmail.com (Y.K.); 3 Department of Nutrition, Assuta Medical Center, Tel Aviv 69710, Israel; 4 Assia Medical Group, Assuta Medical Center, Tel Aviv 69710, Israel; aszold@assia.co.il (A.S.); pplanger@gmail.com (P.L.); doctor@asnatraziel.com (A.R.); sakranas@gmail.com (N.S.); david.goitein@sheba.health.gov.il (D.G.); 5 Department of Health Policy and Management, School of Public Health, Faculty of Health Sciences, Ben-Gurion University of the Negev, Beer-Sheva 84105, Israel; boriskh83@gmail.com (B.K.); motroy@post.bgu.ac.il (Y.M.); 6 Department of Surgery, Holy Family Hospital, Nazareth 16234, Israel; 7 The Azrieli Faculty of Medicine Safed, Bar-Ilan University, Ramat Gan 52900, Israel; 8 Department of Surgery C, Sheba Medical Center, Ramat Gan 52621, Israel; 9 Sackler Faculty of Medicine, Tel-Aviv University, Tel Aviv 69978, Israel

**Keywords:** gastric bypass, small-intestinal bacterial overgrowth, pancreatic exocrine insufficiency, gut microbiota

## Abstract

One anastomosis gastric bypass (OAGB) is an emerging bariatric procedure, yet data on its effect on the gastrointestinal tract are lacking. This study sought to evaluate the incidence of small-intestinal bacterial overgrowth (SIBO) following OAGB; explore its effect on nutritional, gastrointestinal, and weight outcomes; and assess post-OABG occurrence of pancreatic exocrine insufficiency (PEI) and altered gut microbiota composition. A prospective pilot cohort study of patients who underwent primary-OAGB surgery is here reported. The pre-surgical and 6-months-post-surgery measurements included anthropometrics, glucose breath-tests, biochemical tests, gastrointestinal symptoms, quality-of-life, dietary intake, and fecal sample collection. Thirty-two patients (50% females, 44.5 ± 12.3 years) participated in this study, and 29 attended the 6-month follow-up visit. The mean excess weight loss at 6 months post-OAGB was 67.8 ± 21.2%. The glucose breath-test was negative in all pre-surgery and positive in 37.0% at 6 months (*p* = 0.004). Positive glucose breath-test was associated with lower reported dietary intake and folate levels and higher vitamin A deficiency rates (*p* ≤ 0.036). Fecal elastase-1 test (FE1) was negative for all pre-surgery and positive in 26.1% at 6 months (*p* = 0.500). Both alpha and beta diversity decreased at 6 months post-surgery compared to pre-surgery (*p* ≤ 0.026). Relatively high incidences of SIBO and PEI were observed at 6 months post-OAGB, which may explain some gastrointestinal symptoms and nutritional deficiencies.

## 1. Introduction

One anastomosis gastric bypass (OAGB), an emerging bariatric surgery (BS) [1,2], employs a long narrow-sleeve gastric tube in conjunction with end-to-side or side-to-side gastrojejunostomy performed 150–200 cm distal to the ligament of Treitz [2,3]. This surgical technique was found to be effective in terms of weight loss and co-morbidities improvements [2]. However, some areas of concern remain, including substantial gastrointestinal (GI) symptoms [4].

Altered bowel anatomy and motility caused by OAGB could create a blind intestinal loop resulting in small-intestinal bacterial overgrowth (SIBO) [5,6,7,8]. SIBO, characterized by the presence of excessive bacteria in the small intestine [9], is related to the presence of symptoms such as bloating, diarrhea, and gas, but it may also be asymptomatic [9,10]. The traditional “gold standard” for diagnosing SIBO is a culture of the intestinal fluid, but this method has several technical and definition hurdles [8,9,11]. The breath test, a non-invasive test that detects the presence of exhaled hydrogen (H_2_) or methane [9,11] following the ingestion of a carbohydrate substrate, is considered a valid, inexpensive, and safe diagnostic test [12]. Presently, there is no standardized protocol for SIBO assessment in patients who underwent BS [8], and data exploring the prevalence and implications of SIBO following BS are limited [6,13,14,15,16,17,18].

Pancreatic exocrine insufficiency (PEI), a well-known complication after upper GI surgery [19,20,21], refers to an insufficient secretion of pancreatic enzymes and/or sodium bicarbonate that prevents normal digestion [22]. PEI symptoms may include steatorrhea, abdominal pain, and flatulence [19,20,22,23]. Fecal elastase-1 (FE1) serves as a non-invasive marker of pancreatic secretion, and its measurement is considered a relatively reliable diagnostic test [19,20,24]. Notably, pancreatic exocrine function was evaluated only in a limited number of studies among BS patients [19,23,24].

Different studies on various BS techniques have shown that these surgeries induce drastic changes in gut microbiota composition [25,26,27]. Currently, most studies reported on microbiota composition changes following Roux-en-Y gastric bypass (RYGB), sleeve gastrectomy (SG), adjustable gastric banding (AGB), and vertical banded gastroplasty (VBG) [25,26,27,28,29]. However, to the best of our knowledge, no study has investigated these changes following OAGB. 

Therefore, the primary aim of this study was to evaluate the incidence of SIBO following OAGB and to explore its effect on nutritional deficiencies, GI symptoms, and weight loss. The secondary aims were to evaluate PEI incidence and to elucidate alterations in the gut microbiota composition at 6 months following OAGB. 

## 2. Materials and Methods

### 2.1. Study Population

A pilot prospective cohort study included 32 patients who underwent primary-OAGB surgery at the Assuta Medical Centers from October 2018 to March 2020. Inclusion criteria were age between 18 and 65 years, body mass index (BMI) ≥ 40 kg/m^2^ or BMI ≥ 35 kg/m^2^ with co-morbidities [30], and approval of the Assuta Medical Centers BS committee to undergo BS and primary-OAGB surgery. Exclusion criteria included the use of antibiotics [9,12] or probiotics in the preceding month, concomitant use of promotility drugs and laxatives one week prior to each study examination [9,12], uncontrolled mental illness or cognitive deterioration, chronic medical conditions that could interfere with the study (e.g., active cancer, organ-transplant subjects, and advanced kidney disease), treatment with insulin, excessive alcohol consumption, and pregnancy/lactation. 

The study was approved by the Assuta Medical Center Institutional Ethics Committee (#0014-18-ASMC), and informed consent was obtained from all participants.

All patients received the standard of care during the study period [30,31]. The manuscript was written according to the STROBE cohort checklist [32]. 

### 2.2. Pre- and Post-Surgical Follow-Up Evaluations (Baseline and 6 Months)

#### 2.2.1. Medical History

An interview at both time-points collected data on smoking habits and the use of medications and supplements. 

#### 2.2.2. Anthropometric Measurements

Height was measured without shoes, with a wall-mounted stadiometer; weight was measured with a high-capacity weigh-scale; and BMI was calculated and expressed in kg/m^2^. Waist circumference (WC) was measured twice at the umbilicus level [33], and neck circumference (NC) was measured twice, with head erect and eyes facing forward, at the level of the cricothyroid membrane [34]. Excess weight loss (EWL) percentage was calculated as previously recommended [35].

#### 2.2.3. Glucose Breath-Test to Assess SIBO

The test protocol for the breath-test to assess SIBO was performed in accordance with a recently published North American consensus regarding breath-testing in GI disorders [12], but with slight modifications [36]. The breath-test was initiated by collecting a baseline sample where the H_2_ levels in patient’s breath were examined by a breath analyzer (Gastro+™ Gastrolyzer^®^, coVita, Santa Barbara, CA, USA). Then the patients ingested a dose of 50 gr glucose mixed with one cup of water (250 mL) [36]. The participants exhaled into a breath analyzer every 30 min for 2 h, while H_2_ was measured in expired air. A rise in H_2_ of ≥20 parts per million (ppm) during the test when compared with the basal value was considered indicative of SIBO [12]. Patients with SIBO were divided to “early risers” or “late risers” (i.e., the rise in H_2_ of ≥20 ppm occurred within 60 min or after 60 min, respectively). This cutoff was based on the observation that the median oral–cecal transit time was 60 min following RYGB [8]. 

A trained physician performed all assays, and the preparation for the test was verified for each participant with the research team. The test was performed under standard conditions, including a low-carbohydrate diet the day prior to testing and at least 12 h of fasting [9,12]. On the day of testing, smoking was asked to be avoided, and physical activity was asked to be limited during the testing period [9,12]. 

#### 2.2.4. Assessment of Gastrointestinal Symptoms

Data on GI symptoms were collected by an interview, and all participants filled in the Irritable Bowel Syndrome Rome III Diagnostic Criteria questionnaire [37].

#### 2.2.5. Biochemical Tests

Each participant underwent biochemical testing following a 12 h fast, including complete blood count, albumin, total protein, transferrin, ferritin, iron, vitamin B12, folic acid, vitamin A, and vitamin D. Nutritional abnormalities were defined as a plasma level below/above the recommended reference range (Table 1). 

#### 2.2.6. Quality-of-Life Assessment

Quality of life (QoL) was assessed by the Moorehead–Ardelt QoL Questionnaire II (M-A QoLII), which includes 6 key areas: self-esteem, physical activity, social contacts, satisfaction concerning work, pleasure related to sexuality, and eating behavior [38,39,40]. In addition, patients were asked to rate their overall state-of-health from 0 to 100, using a visual analog scale QoL (VAS QoL), with 100 reflecting the “best imaginable state-of-health” [41]. 

#### 2.2.7. Dietary Intake Assessment

Dietary intake in the last month before testing was evaluated by using a food frequency questionnaire (FFQ) [42] that was modified for the current study. The nutrient content of the food items was obtained from the Israeli nutritional software “Zameret” [43].

#### 2.2.8. Physical Activity

Data on physical activity in the last month before testing were collected by an interview. Weekly hours spent in physical activity were calculated by the number of training sessions per week × the duration of exercise in hours.

#### 2.2.9. Fecal Sample Collection and Analysis

Stool samples were self-collected in sterile tubes and a regular FLOQSwabs^®^ transport system (COPAN ITALIA spa, Brescia, Italy) given to the participants in advance at baseline and at 6 months post-surgery and stored at −80 °C until processed and analyzed. For the diagnosis of PEI, samples with a concentration of FE1 of >200 µg/g stool were considered normal. Samples that demonstrated the concentrations ranging from 200 to 100 µg/g stool were considered as exhibiting mild-to-moderate PEI, and <100 µg/g stool were considered as exhibiting severe PEI [21]. 16S amplicon sequencing and microbiome analyses were undertaken, including comparisons of relative abundance at the phylum and genus levels, comparisons of alpha and beta diversity, and differential abundance analysis. Fecal-sample collection and analysis are further detailed in Supplementary Materials Methods S1 [44,45,46,47,48,49]. 

### 2.3. Statistical Methods

The IBM SPSS Statistics for Windows, version 26 (IBM Corp., Armonk, N.Y., USA) was used for statistical analyses. Descriptive statistics were used to describe the distribution of variables associated with characteristics of the study sample. Continuous variables were presented as means ± SD and categorical variables as proportions. Continuous variables that failed the normality test were analyzed by using nonparametric tests.

To compare continuous variables between two time-points, the t-test for dependent groups was performed, and for dichotomous variables, the McNemar test was performed. To test differences in continuous variables between two groups, the independent-samples t-test was performed. For comparison of categorical variables, the Chi-Square test or Fisher’s exact test was performed. The level of significance for all analyses was set at *p* < 0.05. For the statistical methods used for the comparison of the results of microbiota analyses, see Supplementary Materials Methods S1.

## 3. Results

### 3.1. Characteristics of the Study Participants at Baseline and at 6 Months Post-Surgery

Thirty-two patients (50% females) who underwent primary OAGB surgery participated in this study, and 29 of them attended the 6-month follow-up visit (range: 5.6–7.1 months). One patient underwent BS in another hospital, one cancelled the surgery, and one withdrew from the study. Their mean age and BMI pre-surgery were 44.5 ± 12.3 years (range: 18–62 years) and 41.7 ± 6.6 kg/m^2^ (range: 33.0–62.2 kg/m^2^), respectively. The mean length of the bypassed limb was 176.3 ± 21.9 cm (range: 150–200 cm).

**Figure 1:**
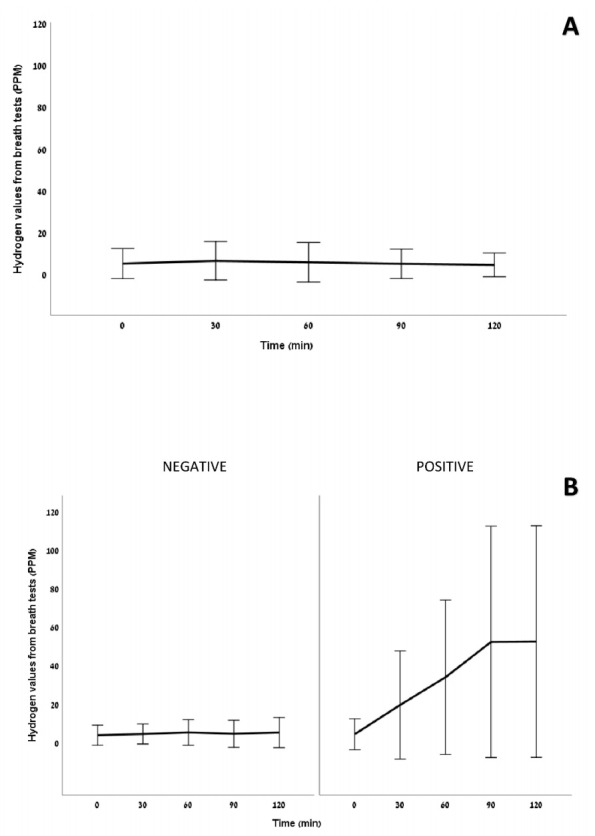
Mean (±SD) glucose breath-test results (**A**) at baseline and (**B**) at 6 months post-surgery. Abbreviations: parts per million (PPM); hydrogen (H_2_). In Figure 1A, H_2_ values for all at 0 min were 4.6 ± 3.6 (range: 1–15). In Figure 1B, H_2_ values for all at 0 min were 3.5 ± 3.1 (range: 1–13); a rise in H_2_ of ≥20 ppm during the test when compared with the basal value considered as indicative of SIBO; Among the patients with a positive glucose breath-test, 6 (60%) were “early risers” (i.e., H_2_ of ≥20 ppm rise occurred up to 60 min), and 4 (40%) were “late risers” (i.e., H_2_ of ≥20 ppm rise occurred after 60 min).

At 6 months post-surgery, %EWL was 67.8 ± 21.2%, patients reported higher QoL scores, hemoglobin was significantly reduced, patients reported significantly lower dietary intake and higher rates of flatulence, frequent soft stool, and hair-loss when compared to baseline (*p* ≤ 0.021 for all) (Table 1). The glucose breath-test was negative in all patients at baseline and positive in 10/27 (37.0%) patients at 6 months post-surgery (*p* = 0.004) (Table 1). Figure 1A,B presents the mean glucose breath-test results at baseline and 6 months post-surgery, respectively.

The FE1 test was negative for all patients at baseline (*n* = 12) and positive in 6/23 (26.1%) patients at 6 months post-surgery (*p* = 0.500) (Table 1).

There were no re-hospitalizations during the study follow-up term, but two patients reported emergency-room visits due to high fever and abdominal pain.

### 3.2. Comparison between Patients According to Glucose Breath-Test Results at 6 Months Post-Surgery

No significant difference was found between patients with positive and negative glucose breath-tests at baseline. A comparison between patients according to their glucose breath-test results at 6 months post-surgery is presented in Table 2.

The reported dietary intake was significantly lower among patients with a positive glucose breath-test (*p* ≤ 0.036). Lower levels of folate (8.4 ± 3.6 vs. 14.7 ± 5.5 ng/mL, *p* = 0.003) and higher vitamin A deficiency rates (40 vs. 0%, *p* = 0.014) were found among the positive glucose breath-test group, although no significant difference in supplement use was found between both groups. Regurgitation was significantly more common among the positive glucose breath-test group (30 vs. 0%, *p* = 0.041) (Table 2).

### 3.3. Gut Microbiota Analysis

The phyla Proteobacteria and Verrucomicrobia showed significant increases in relative abundance over time, whereas Firmicutes, Fusobacteria, and Tenericutes showed significant decreases in relative abundance over time for all patients.

**Figure 2:**
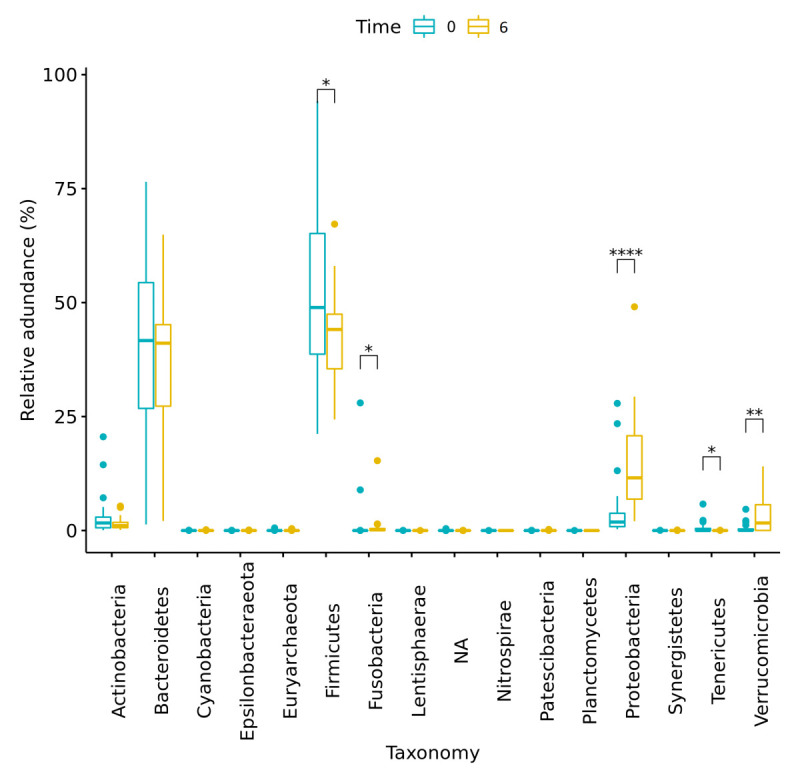
Changes in relative abundances of phyla for all patients (*n* = 28) from baseline (Time 0) to 6 months post-surgery (Time 6). Significant differences in abundance are marked with an asterisk (* indicates *p* < 0.05; ** indicates *p* < 0.005; **** indicates *p* < 0.00005). NA = sequences that were assigned to kingdom bacteria but were not assigned to a specific phylum.

**Figure 3:**
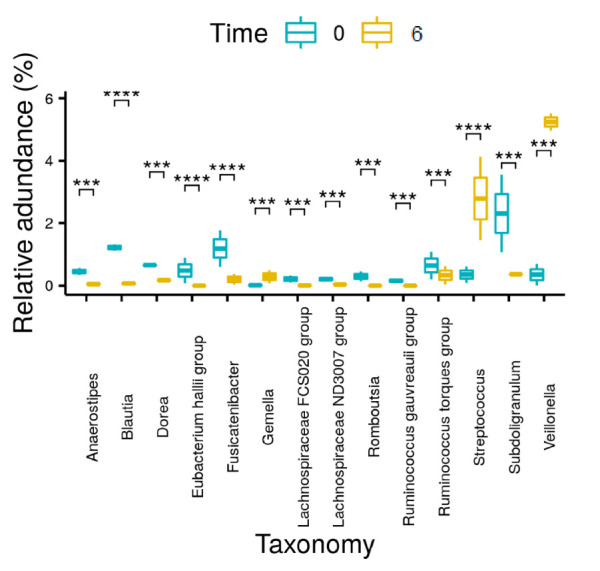
Changes in relative abundances of genera for all patients (*n* = 28) from baseline (Time 0) to 6 months post-surgery (Time 6). Significant differences in abundance are marked with an asterisk (*** indicates *p* < 0.0005; **** indicates *p* < 0.00005). Note that only significant differences in abundance with *p* < 0.0005 are presented here.

Changes in the relative abundance of main phyla over time are presented in Figure 2 and Table 3. Changes in the relative abundance of main genera over time are presented in Figure 3 and Supplementary Materials Table S1. Moreover, significant changes were observed in the differential abundance analysis at the genera level, using LefSe (Supplementary Materials Figure S1 and Table S2).

**Figure 4:**
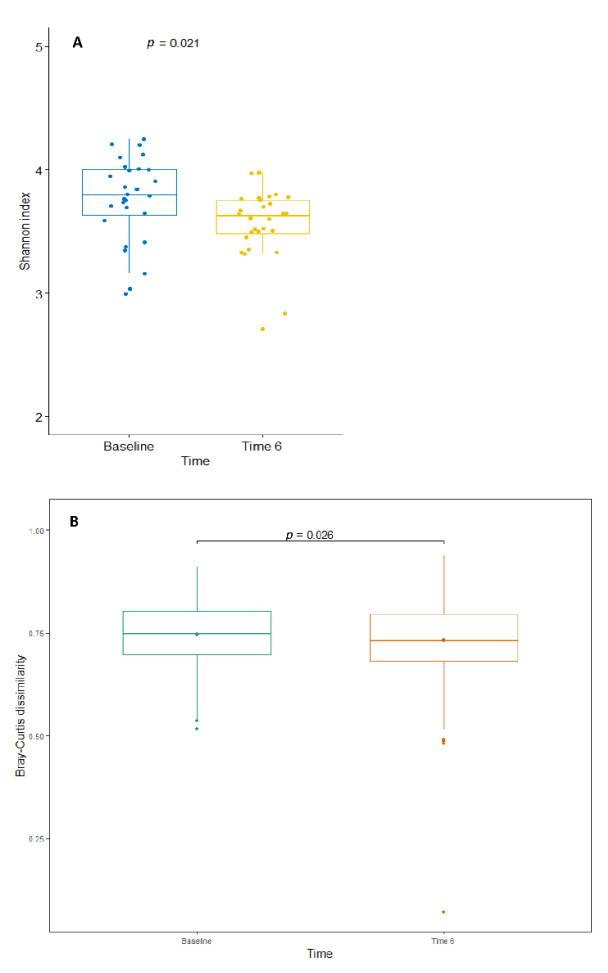
(**A**) Alpha diversity (using the Shannon index) and (**B**) beta diversity (using the Bray–Curtis dissimilarity metric) for all patients (*n* = 28) from baseline (Time 0) to 6 months post-surgery (Time 6).

Significant reductions in alpha diversity (Shannon index, *p* = 0.021) and beta diversity (Bray–Curtis dissimilarity index, *p* = 0.026) over time were observed among all patients (Figure 4A,B, respectively).

### 3.4. Gut Microbiota Analysis According to Glucose Breath-Test Results at 6 Months Post-Surgery

**Figure 5:**
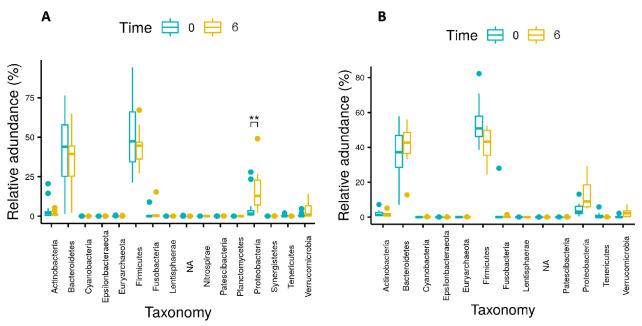
Changes in relative abundances of phyla for (**A**) patients (*n* = 17) who did not develop SIBO and (**B**) patients (*n* = 10) who developed SIBO from baseline (Time 0) to 6 months post-surgery (Time 6). Significant differences in abundance are marked with an asterisk (** indicates *p* < 0.005). NA = sequences that were assigned to the kingdom bacteria but were not assigned to a specific phylum.

The changes in the relative abundance of main phyla for patients who did not develop SIBO and patients who developed SIBO over time are presented in Figure 5A,B, respectively. Significant changes were observed in the differential abundance analysis at the genera level, using LefSe, for both groups (Supplementary Materials Figures S2 and S3 and Tables S3 and S4). A comparison of the results from the differential abundances at the genera level, using LefSe, for both patient groups is presented in Supplementary Materials Table S5.

No significant differences in alpha diversity were observed between SIBO groups at baseline (*p* = 0.98) and at 6 months post-surgery (*p* = 0.675). A significant reduction in alpha diversity was observed over time within the group that did not develop SIBO at 6 months post-surgery (Shannon index, *p* = 0.038), while no significant reduction in alpha diversity over time was observed within the group that developed SIBO at 6 months post-surgery (*p* = 0.46) (Supplementary Materials Figure S4). Significant differences in beta diversity between SIBO groups were observed at baseline (Unweighted Unifrac, *p* = 0.033), but not at 6 months post-surgery (*p* = 0.75). There was a significant reduction in beta diversity over time within the group that did not develop SIBO at 6 months post-surgery (Unweighted Unifrac, *p* = 0.0018), while no significant change over time was observed within the group that developed SIBO at 6 months post-surgery (*p* = 0.79) (Supplementary Materials Figure S5). Moreover, the magnitude of change over time was lower among the group that developed SIBO at 6 months post-surgery (Unweighted Unifrac, *p* = 0.0055) (Supplementary Materials Figure S6).

### 3.5. Gut Microbiota Analysis of Patients According to FE1 Test at 6 Months Post-Surgery

Significantly higher beta diversity was observed in patients who presented a positive FE1 test at 6 months post-surgery (Unweighted Unifrac, *p* = 0.0034) (Supplementary Materials Figure S7). However, no difference was noticed for changes in alpha diversity between these groups.

## 4. Discussion

In the present study, we report the incidence of SIBO and PEI and the corresponding changes in the gut microbiota composition during a 6-month follow-up period after OAGB.

A substantial improvement in anthropometric parameters was found among our participants at 6 months post-surgery, accompanied by a high satisfaction rate from overall state-of-health and QoL. However, more than a third of the patients developed SIBO, and more than a quarter were diagnosed with PEI at 6 months following OAGB. An overlap of both conditions was found in two patients. Moreover, relatively high rates of frequent soft stools and flatulence were reported at 6 months post-surgery.

Data on the prevalence and implications of SIBO following BS are currently scarce [6,13,14,15,16,17,18]. Furthermore, most published studies lacked standardization. Our results are in line with a previous study that found a prevalence of 40% SIBO in patients with a median follow-up of 9.2 months after RYGB, although the prevalence of SIBO was 15% at baseline among these subjects [18], as compared to 0% in the present study. However, two other studies that investigated this phenomenon included only patients with a history of RYGB, OAGB, and SG, with subjective abdominal symptoms, and found much higher rates of SIBO [6,17]. Therefore, it may be reasonable to recommend a routine workup for SIBO in patients with a history of BS in the context of abdominal symptoms [6]. Moreover, the management of SIBO can be challenging following BS [50].

In the current study, there were several significant differences between patients with SIBO when compared to those without SIBO at 6 months post-surgery, including higher vitamin A deficiency rates, lower folate levels, a trend toward lower vitamin B12 levels, higher reported rates of regurgitation, and lower dietary intake among the group that developed SIBO. It is important to mention that no difference was found for these parameters between the groups at baseline, except vitamin A, which was not measured. However, the prevalence of vitamin A deficiency among BS candidates before surgery is presumably low [51]. Moreover, high and comparable adherence to supplementation was found in both groups at 6 months post-surgery. In agreement with our results, established SIBO is commonly related to fat-soluble vitamins and vitamin B12 deficiencies [8,9]. However, in contrast to our findings, SIBO is generally related to excessive folate levels secondary to bacterial synthesis [8,9]. One plausible explanation for our findings can be the trend towards increased folic acid supplementation among the group that did not develop SIBO at 6 months post-surgery. However, folic acid can also be found in multivitamin, iron, and vitamin B12 supplementation.

The adverse nutritional consequences of SIBO may involve multiple factors, including diminished food intake due to the presence of GI symptoms [9]. In the current study, patients who developed SIBO indeed reported significantly lower dietary intake at 6 months post-surgery, but no difference in GI symptoms, expect regurgitation, was reported, and no differences in anthropometric parameters were observed between the groups at 6 months post-surgery.

PEI was found in more than a quarter of the participants at 6 months post-surgery, but in none of them at baseline. This rate is higher than in a previous study of 22 patients one year following RYGB and OAGB that reported a rate of 9.1% PEI according to FE1 [23], but similar to a study of 188 RYGB patients with a mean follow-up of 12.5 months which found a prevalence of 31% PEI according to FE1 [19]. The latter study also found that a shorter biliopancreatic limb length lessened the prevalence of PEI [19]. Here, there was no difference in the bypassed limb length between the PEI and non-PEI groups at 6 months post-surgery. Pancreatic enzyme replacement therapy has been advocated for patients with symptomatic PEI, in addition to the implementation of the needed dietary modifications [21]. However, since the purpose of BS is to achieve weight loss, it is still uncertain what is the optimal therapy in such cases, and more research is currently needed [21], yet one study showed that pancreatic enzyme replacement therapy did not affect weight loss during three months of treatment [52].

The analysis of the gut microbiota in our cohort resulted in several observations. An overall reduction in both the richness and diversity of the microbiota was evident 6 months following OAGB. These results contrast with the current literature regarding alpha diversity changes in the short term, following different types of bariatric procedures [25]. Nonetheless, in the current study, the microbiota composition in terms of similarity between samples increased over time. In addition, significant changes in abundances of specific phyla and genera were observed over time. Proteobacteria, which may be beneficial due to a decrease in systemic inflammation and improved glucose homeostasis [25], and Verrucomicrobia, including *Akkermansia*, which are negatively related to the desire to eat sweets [26], were significantly increased. Although the Bacteroidetes phylum showed a trend towards decrease, its related genera *Bacteroides* and *Prevotella* significantly increased, and *Alistipes* significantly decreased. *Bacteroides* and *Alistipes* are positively related to a reduction of body fat mass and leptin, which affects the reduction of body weight [26,29]. *Bacteroides* is also related to remission of type 2 diabetes [27]. Actinobacteria showed a trend of decrease; Firmicutes and related genera, including *Blautia*, *Dorea,* and *Faecalibacterium* significantly decreased; and *Roseburia* and *Streptococcus* significantly increased. *Blautia* and *Dorea* have been suggested to positively correlate with leptin levels [26]. Our study is only partially in line with previous studies of RYGB patients which showed an increase in *Bacteroides* and *Alistipes* (Bacteroidetes) and *Escherichia* (Proteobacteria), and a decrease in *Dorea*, *Blautia*, and *Roseburia* (Firmicutes) and Actinobacteria at 6 months post-surgery [25,26,27,29].

To the best of our knowledge, no studies have yet investigated the associations between SIBO and the microbiota among BS patients. In the current study, for both SIBO and non-SIBO patients, a reduction in alpha diversity was noted; however, these results were significant only for the non-SIBO group and are thus probably unrelated to SIBO.

Patients who had a positive PEI diagnosis at 6 months post-surgery exhibited a significantly higher beta diversity in comparison to non-PEI. This result is in line with a large-scale population-based study that found that exocrine pancreatic function is associated with the microbiota composition and diversity [53]. Nonetheless, the exact mechanisms that link both and the pathophysiological consequences are currently unknown.

The strengths of the current study include its novelty, as this is the first study to examine SIBO, PEI, and microbiota dynamics following OAGB, in depth. Nevertheless, some limitations are noteworthy. First, our pilot study included a small sample size. Thus, the lack of significant differences between groups might be a consequence of its being underpowered. However, the sample power calculated with no-SIBO cases at baseline and 37% over time was 0.999. Second, over-diagnosis of SIBO may occur following gastric bypass surgeries, according to the traditional cutoff time for positive SIBO diagnosis. By this period of time, the carbohydrate substrate may have already reached the colon in some patients [8,17]. However, a majority of the patients who developed SIBO in our cohort were “early risers”, and this is believed to reflect abnormal fermentation of the carbohydrate substrate in the small intestine [7]. Moreover, breath-tests were performed after the ingestion of glucose, which, compared to lactulose, generally shows a single “early” peak of hydrogen excretion [36]. In addition, the FE1 test was not available for all participants for a pair-wise assessment. Therefore, future studies of larger sample size are needed to corroborate these findings with respect to SIBO and PEI in the long-term, following OAGB. Future studies may also include an analysis of inflammatory markers to assess microbiome changes and possible systemic effects.

## 5. Conclusions

The relatively high incidence rates of SIBO and PEI may partially explain the GI symptoms and nutritional deficiencies found following OAGB. These findings were accompanied by a less diverse and increasingly similar microbiota composition at 6 months post-surgery, coupled with significant changes in abundances of specific phyla and genera over time. These findings reflect on the pathophysiology of the adverse effects of OABG and should allow future guidance on the diagnosis and treatment of GI symptoms following OAGB.

## Figures and Tables

**Table 1:** Characteristics of the study participants at baseline and at 6 months post-surgery.

Variable ^a^	Baseline (*n* = 32)	6 Months Post-Surgery (*n* = 29)	*p*-Value
Age (years)	44.5 ± 12.3	-	-
Sex (%female)	50	-	-
Marital Status (%)			
Married	75	-	-
Divorced	6.3	-	-
Single	18.8	-	-
Co-morbidities (%)			
Diabetes	9.4	-	-
IFG	50	-	-
NAFLD	84.4	-	-
Dyslipidemia	59.4	-	-
Hypertension	21.9	-	-
Sleep Apnea	15.6	-	-
Hypothyroidism	3.1	-	-
Gastroesophageal Reflux Disease	21.9	-	-
Orthopedic Problems	31.3	-	-
Medication (%)			
Drugs for diabetes	6.3	0	0.500
Drugs for dyslipidemia	12.5	3.4	0.500
Drugs for Hypertension	18.8	10.3	0.500
Anti-aggregation drugs	6.3	3.4	1.000
Drugs for hypothyroidism	3.1	3.4	1.000
Antacids	9.4	31	0.109
Anti-depressive drugs	3.1	3.4	1.000
Anthropometrics			
Weight (kg)	120.8 ± 25.2	90.9 ± 20.3	<0.001
Height (meter)	1.7 ± 0.1	-	-
BMI (kg/m^2^)	41.7 ± 6.6	31.4 ± 5.6	<0.001
WC (cm)	122.8 ± 14.3	100.5 ± 13.9	<0.001
NC (cm)	38.7 ± 4.5	34.8 ± 3.7	<0.001
%EWL	NR	67.8 ± 21.2	-
Lifestyle			
Smoking (%yes)	6.3	6.9	1.000
Physical activity (%yes)	56.3	79.3	0.180
Physical activity (h/week)	1.2 ± 1.6	2.8 ± 2.3	0.017
Dietary intake			
Calories (kcal/day)	2563.0 ± 979.6	1626.7 ± 712.8	<0.001
Protein (g/day)	136.7 ± 48.8	87.3 ± 40.2	<0.001
Carbohydrates (g/day)	226.7 ± 85.2	150.0 ± 73.7	<0.001
Fats (g/day)	116.8 ± 63.4	69.5 ± 38.4	<0.001
Percent of food intake compared to before surgery	NR	31.7 ± 14.6	-
No. of dietitian appointments after surgery	NR	4.3 ± 3.5	-
Participation in support group after surgery (%yes)	NR	13.8	-
Supplementation (%)			
Multivitamin	43.8	93.1	<0.001
Calcium	6.3	62.1	<0.001
Vitamin D	62.5	86.2	0.039
Vitamin B12	34.4	72.4	0.007
Iron	18.8	24.1	0.375
Folic Acid	18.8	6.9	0.250
Biochemical tests ᵇ			
Hemoglobin (g/dL)	14.0 ± 1.3	13.2 ± 1.2	<0.001
%anemia (<13.5(male), <12(female))	12.5	28.6	0.063
MCV (fL)	83.3 ± 4.7	86.0 ± 5.1	<0.001
%low values (<80)	25.0	10.7	0.063
%high values (>95)	0	0	NR
MCHC (g/dL)	33.8 ± 0.7	33.6 ± 0.7	0.632
%low values (<33)	6.3	14.3	0.625
%high values (>37)	0	0	NR
Albumin (g/dL)	4.4 ± 0.3	4.2 ± 0.3	0.002
%hypoalbuminemia (<3.5)	0	3.6	1.000
Total protein (g/dL)	7.6 ± 0.4	7.2 ± 0.4	<0.001
%low values (<6.3)	0	0	NR
Iron (µg/dL)	90.3 ± 29.8	80.6 ± 25.1	0.107
%deficiency (<49 [male], <37 [female])	0	3.7	1.000
Ferritin (ng/mL)	155.0 ± 138.3	154.4 ± 125.3	0.564
%deficiency (<22[male], <10[female])	0	0	NR
Transferrin (mg/dL)	277.7 ± 37.5	238.9 ± 55.7	0.001
%low values (<220)	6.3	29.6	0.070
%high values (>400)	0	0	NR
Transferrin saturation (%)	23.8 ± 8.8	26.4 ± 14.1	0.323
%low values (<20)	43.8	33.3	0.289
Folate (ng/mL)	11.0 ± 5.6	12.4 ± 5.7	0.285
%deficiency (<2.76)	0	0	NR
Vitamin B12 (pg/mL)	519.5 ± 233.1	534.6 ± 221.9	0.725
%deficiency (<239)	0	3.7	1.000
Vitamin D (ng/mL)	25.3 ± 8.6	27.5 ± 10.2	0.366
%insufficiency (<30)	78.1	63.0	0.289
%deficiency (<20)	25.0	18.5	1.000
Vitamin A (µg/dL)	-	42.4 ± 10.1	-
%deficiency (<30)	-	15.4	-
Quality of life			
VAS QoL	63.8 ± 18.3	81.0 ± 16.0	<0.001
M-A QoLII score ^c^	1.1 ± 0.8	1.8 ± 0.9	0.002
M-A QoLII (%Good/Very good) ^d^	56.3	82.8	0.065
GI symptoms (%)			
ROME III score (%positive) ^e^	9.4	20.7	0.250
Vomit	0	0	NR
Nausea	6.3	24.1	0.125
Regurgitation	9.4	10.3	1.000
Hiccups	9.4	31.0	0.070
Heartburn	28.1	10.3	0.070
Abdominal pain	-	17.2	-
Flatulence	12.5	58.6	0.002
Frequent soft stool	6.3	34.5	0.021
No. of feces per day	1.5 ± 0.8	1.7 ± 1.2	0.188
≥3 feces per day	3.1	13.8	0.250
Hair loss	21.9	48.3	0.016
Glucose breath test (%positive) ^f,g^	0	37.0	0.004
PEI (%positive) ^g,h^	0	26.1 ^i^	0.500

Abbreviations: body mass index (BMI), excess weight loss (EWL), gastrointestinal (GI), impaired fasting glucose (IFG), mean cell hemoglobin concentration (MCHC), mean cell volume (MCV), Moorehead–Ardelt Quality of Life Questionnaire II (M-A QoLII); neck circumference (NC), nonalcoholic fatty liver disease (NAFLD), not relevant (NR), pancreatic exocrine insufficiency (PEI), visual analogue scale quality of life (VAS QoL), waist circumference (WC). ^a^ Values expressed as the mean ± standard deviation, unless otherwise stated. ^b^ *n* = 32 for this test at baseline and *n* = 27 for this test at 6 months post-surgery. ^c^ A 10-point Likert scale is used for scoring, and its total score ranges from −3 to +3 (very poor to very good quality of life). ^d^ Score of 1.1–2 equals “good quality of life”, and 2.1–3 equals “very good quality of life”. ^e^ Irritable Bowel Syndrome was considered present when abdominal pain occurred more than 2 to 3 days a month, relieved after defecation, was related to changes in form and frequency of defecation and existed for 6 months or more. ^f^ *n* = 31 for this test at baseline and *n* = 27 for this test at 6 months post-surgery. ^g^ There was no difference in bypassed limb length between patients with positive and negative glucose breath test or FE1 test. ^h^ *n* = 12 for this test at baseline, and *n* = 23 for this test at 6 months post-surgery. ^i^ Four patients were categorized with severe PEI (FE1 < 100 µg/g), and 2 patients with mild-to-moderate PEI (FE1 = 100–200 µg/g).

**Table 2:** Comparison between patients according to their glucose breath-test results at 6 months post-surgery ^a^.

Variable ^b^	SIBO Positive (*n* = 10)	SIBO Negative (*n* = 17)	*p*-Value
Age (years)	41.0 ± 9.9	48.3 ± 12.8	0.133
Sex (%female)	80	41.2	0.107
Medication (%)			
Drugs for diabetes	0	0	NR
Drugs for dyslipidemia	0	5.9	1.000
Drugs for Hypertension	0	17.6	0.274
Anti-aggregates	0	5.9	1.000
Drugs for hypothyroidism	0	5.9	1.000
Anti-acids	20	35.3	0.666
Drugs for depression	10	0	0.370
Bypass length (cm)	175.0 ± 26.4	175.3 ± 19.7	0.976
Anthropometric measurements			
Weight (kg)	87.2 ± 15.2	93.7 ± 23.8	0.449
BMI (kg/m^2^)	32.0 ± 5.4	31.2 ± 6.1	0.755
WC (cm)	97.2 ± 9.3	102.3 ± 16.2	0.370
NC (cm)	33.3 ± 3.2	35.5 ± 4.0	0.158
%EWL	64.8 ± 19.6	69.4 ± 22.5	0.600
Lifestyle			
Smoking (%yes)	10	5.9	1.000
Physical Activity (%yes)	80	82.4	1.000
Physical Activity (h/week)	2.7 ± 2.3	2.9 ± 2.4	0.766
Dietary intake			
Calories (kcal/day)	1192.9 ± 471.6	1908.4 ± 704.1	0.009
Protein (g/day)	65.1 ± 26.9	101.3 ± 42.2	0.023
Carbohydrates (g/day)	113.6 ± 52.9	175.1 ± 77.3	0.036
Fats (g/day)	49.2 ± 19.3	82.1 ± 41.8	0.028
Percent of food intake compared to before surgery	33.5 ± 13.1	28.8 ± 13.1	0.414
No. of dietitian appointments after surgery	3.5 ± 1.8	4.6 ± 4.4	0.675
Participation in support group after surgery (%)	10	17.6	1.000
Supplementation (%)			
Multivitamin	90	94.1	1.000
Calcium	60	64.7	1.000
Vitamin D	70	94.1	0.128
Vitamin B12	60	82.4	0.365
Iron	10	29.4	0.363
Folic Acid	0	11.8	0.516
Biochemical tests			
Hemoglobin (g/dL)	13.2 ± 0.4	13.2 ± 1.5	0.928
%anemia (<13.5(male), <12(female))	20	35.3	0.666
MCV (fL)	88.1 ± 3.2	84.9 ± 5.8	0.116
%low values (<80)	0	17.6	0.274
%high values (>95)	0	0	NR
MCHC (g/dL)	33.7 ± 0.7	33.7 ± 0.7	0.891
%low values (<33)	10	11.8	1.000
%high values (>37)	0	0	NR
Albumin (g/dL)	4.2 ± 0.4	4.2 ± 0.3	0.967
%hypoalbuminemia (<3.5)	10	0	0.370
Total protein (g/dL)	7.2 ± 0.4	7.2 ± 0.4	0.994
%low values (<6.3)	0	0	NR
Iron (µg/dL)	75.9 ± 13.4	83.3 ± 30.0	0.470
%deficiency (<49(male), <37(female))	0	5.9	1.000
Ferritin (ng/mL)	98.9 ± 80.4	187.0 ± 137.2	0.077
%deficiency (<22(male), <10(female))	0	0	NR
Transferrin (mg/dL)	249.8 ± 60.3	232.5 ± 53.7	0.477
%low values (<220)	20	35.3	0.666
%high values (>400)	0	0	NR
Transferrin saturation (%)	23.1 ± 6.5	28.3 ± 17.0	0.364
%low values (<20)	40	29.4	0.683
Folate (ng/mL)	8.4 ± 3.6	14.7 ± 5.5	0.003
%deficiency (<2.76)	0	0	NR
Vitamin B12 (pg/mL)	442.1 ± 212.8	589.1 ± 214.6	0.097
%deficiency (<239)	10	0	0.370
Vitamin D (ng/mL)	25.5 ± 10.2	28.7 ± 10.4	0.444
%insufficiency (<30)	60	64.7	1.000
%deficiency (<20)	30	11.8	0.326
Vitamin A (µg/dL)	38.5 ± 12.9	44.9 ± 7.3	0.171
%deficiency (<30)	40	0	0.014
Quality of life			
VAS QoL	77.5 ± 19.5	84.4 ± 9.2	0.317
M-A QoLII score ^c^	1.7 ± 0.7	2.0 ± 0.7	0.265
M-A QoLII (%Good/Very good) ^d^	80	88.2	0.613
GI symptoms (%)			
ROME III score (%positive) ^e^	10	23.5	0.621
Vomit	0	0	NR
Nausea	20	23.5	1.000
Regurgitation	30	0	0.041
Hiccups	40	23.5	0.415
Heartburn	10	11.8	1.000
Abdominal pain	30	11.8	0.326
Flatulence	50	64.7	0.453
Frequent soft stool	50	17.6	0.102
No. of feces per day	1.6 ± 0.7	1.8 ± 1.4	0.598
≥3 feces per day	10	17.6	1.000
Hair loss	70	35.3	0.120
PEI (%positive) ^f^	22.2	23.1	1.000

Abbreviations: body mass index (BMI), excess weight loss (EWL), gastrointestinal (GI), mean cell hemoglobin concentration (MCHC), mean cell volume (MCV), Moorehead–Ardelt Quality of Life Questionnaire II (M-A QoLII), neck circumference (NC), not relevant (NR), pancreatic exocrine insufficiency (PEI), small intestine bacterial overgrowth (SIBO), visual analogue scale quality of life (VAS QoL), waist circumference (WC). ^a^ No significant differences were found between positive SIBO and negative SIBO groups at baseline. ^b^ Values expressed as the mean ± standard deviation, unless otherwise stated. ^c^ A 10-point Likert scale is used for scoring, and its total score ranges from −3 to +3 (very poor to very good quality of life). ^d^ Score of 1.1–2 equals “good quality of life”, and 2.1–3 equals “very good quality of life”. ^e^ Irritable Bowel Syndrome was considered present when abdominal pain occurred more than 2 to 3 days a month, relieved after defecation, was related to changes in form and frequency of defecation, and existed for 6 months or more. ^f^ *n* = 22, additional patient had FE1 test but not glucose breath-test.

**Table 3:** Changes in relative abundances of phyla for all patients from baseline to 6 months post-surgery (*n* = 28). Only significant results (*p* < 0.05) are listed.

Phyla	Relative Abundance at Baseline (%)			Relative Abundance at 6 Months Post-Surgery (%)			*p*-Value (FDR Adjusted)
	Mean	Min	Max	Mean	Min	Max	
Firmicutes	52.28	27.54	86.38	42.47	24.35	67.20	0.021
Fusobacteria	1.320	0	27.99	0.87	0	15.32	0.021
Proteobacteria	4.533	0.288	27.88	14.17	2.046	49.07	<0.001
Tenericutes	0.505	0	5.802	0.003	0	0.061	0.021
Verrucomicrobia	0.446	0	4.659	3.326	0	14.07	0.002

## Data Availability

The data that support the findings of this study are available from the corresponding author, J.M.-G., upon reasonable request. The raw sequencing data were deposited in the European Bioinformatics Institute European Nucleotide Archive under accession no. PRJEB47612.

## Supplementary Materials

The following are available online at https://www.mdpi.com/article/10.3390/nu14020304/s1. Methods S1: Supplementary Methods. Figure S1: Differential abundance analysis at the genera level, using LefSe, for all patients from baseline (Time 0) to 6 months (Time 6) post-surgery (*n* = 28). Figure S2: Differential abundance analysis at the genera level, using LefSe, for patients who did not develop SIBO from baseline (Time 0) to 6 months (Time 6) post-surgery (*n* = 17). Figure S3: Differential abundance analysis at the genera level, using LefSe, for patients who developed SIBO from baseline (Time 0) to 6 months (Time 6) post-surgery (*n* = 10). Figure S4: Differences in alpha diversity (using the Shannon Index). Figure S5: Differences in beta diversity (using the Unweighted Unifrac metric). Figure S6: Differences in the delta of change of beta diversity (using the Unweighted Unifrac metric) over time between the group that did not develop SIBO at 6 months post-surgery (no SIBO at T6, *n* = 17) and the group that developed SIBO at 6 months post-surgery (SIBO at T6, *n* = 10). Figure S7: Differences in beta diversity (using the Unweighted Unifrac metric) when comparing samples according to FE1 test at 6 months post-surgery. Table S1: Changes in relative abundances of genera for all patients from baseline to 6 months post-surgery (*n* = 28). Table S2: Differential abundance analysis at the genera level, using LefSe, for all patients from baseline to 6 months post-surgery (*n* = 28). Table S3: Differential abundance analysis at the genera level, using LefSe, for patients who did not develop SIBO from baseline to 6 months post-surgery (*n* = 17). Table S4: Differential abundance analysis at the genera level, using LefSe, for patients who developed SIBO from baseline to 6 months post-surgery (*n* = 10). Table S5: Comparison of the differential abundance analysis at the genera level, using LefSe results, for patients who did not develop SIBO from baseline to 6 months post-surgery (SIBO neg) and patients who did develop SIBO (SIBO pos).

## Abbreviations

Adjustable gastric banding (AGB), bariatric surgery (BS), body mass index (BMI), excess weight loss (EWL), fecal elastase-1 (FE1), food frequency questionnaire (FFQ), gastrointestinal (GI), hydrogen (H_2_), impaired fasting glucose (IFG), mean cell hemoglobin concentration (MCHC), mean cell volume (MCV), Moorehead–Ardelt Quality of Life Questionnaire II (M-A QoLII), neck circumference (NC), nonalcoholic fatty liver disease (NAFLD), one anastomosis gastric bypass (OAGB), pancreatic exocrine insufficiency (PEI), parts per million (PPM), Roux-en-Y gastric bypass (RYGB), sleeve gastrectomy (SG), small-intestinal bacterial overgrowth (SIBO), vertical banded gastroplasty (VBG), visual analogue scale quality of life (VAS QoL), waist circumference (WC).
