# Supplementary material for: The Effects of One Anastomosis Gastric Bypass Surgery on the Gastrointestinal Tract
Source: Nutrients. 2022 Jan 12;14(2):304. doi: 10.3390/nu14020304 (PMC8778673; doi:10.3390/nu14020304)

Figure S2: Differential abundance analysis at the genera level using LefSe for patients who did not develop SIBO from baseline (Time 0) to 6 months (Time 6) post-surgery (n=17).

Only the top 10 significant results (p<0.05) are presented.

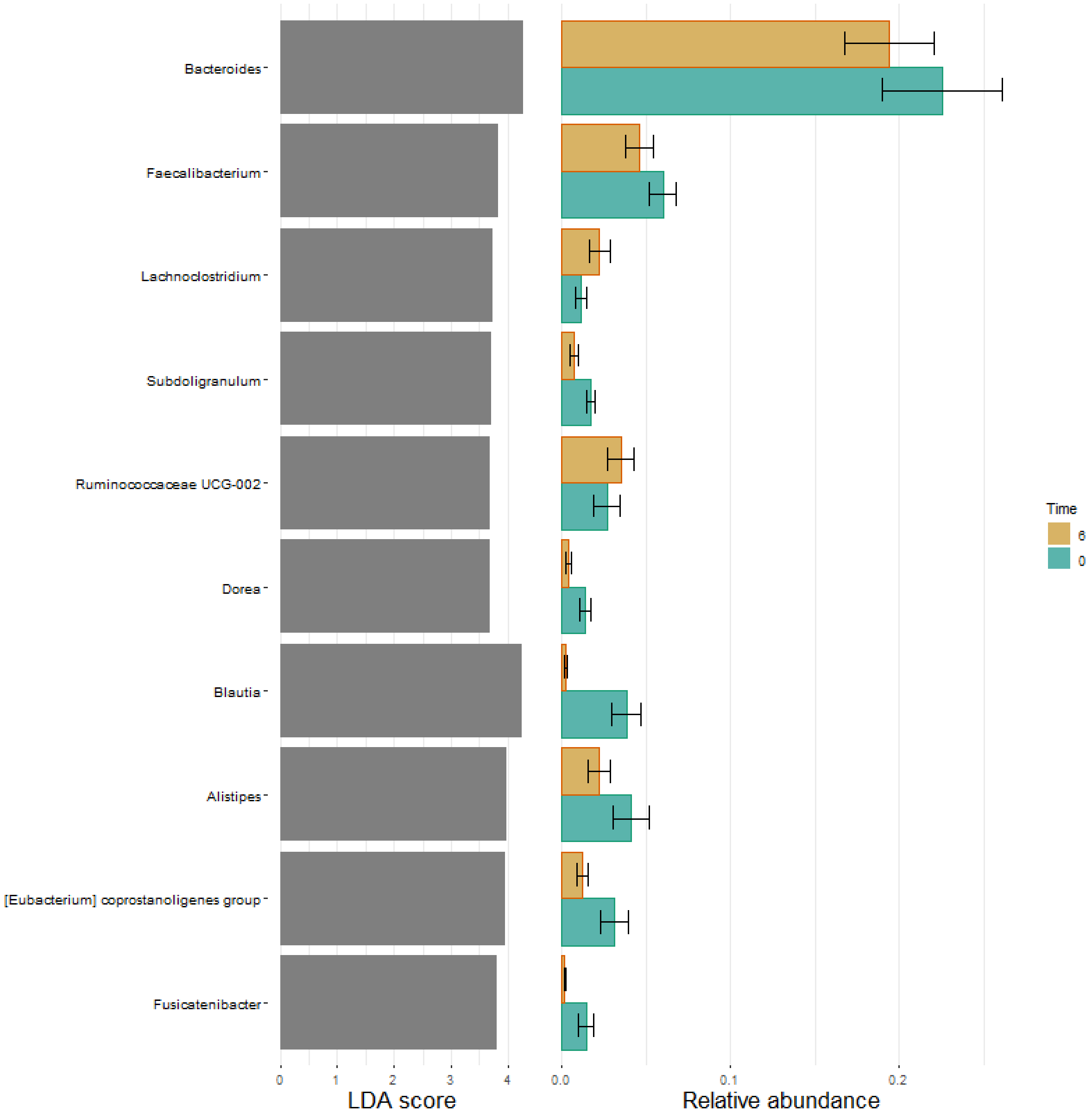

Supplement: Supplementary file 1 [file nutrients-14-00304-s001.zip › Figure S2.pdf]
