# Supplementary material for: The Effects of One Anastomosis Gastric Bypass Surgery on the Gastrointestinal Tract
Source: Nutrients. 2022 Jan 12;14(2):304. doi: 10.3390/nu14020304 (PMC8778673; doi:10.3390/nu14020304)

Figure S3: Differential abundance analysis at the genera level using LefSe for patients who developed SIBO from baseline (Time 0) to 6 months (Time 6) post-surgery (n=10).

Only the top 10 significant results ($p<0.05$) are presented.

NA = Sequences that were assigned at a higher taxonomic level (kingdom, phylum, class, order, family) but were not assigned to a specific genus.

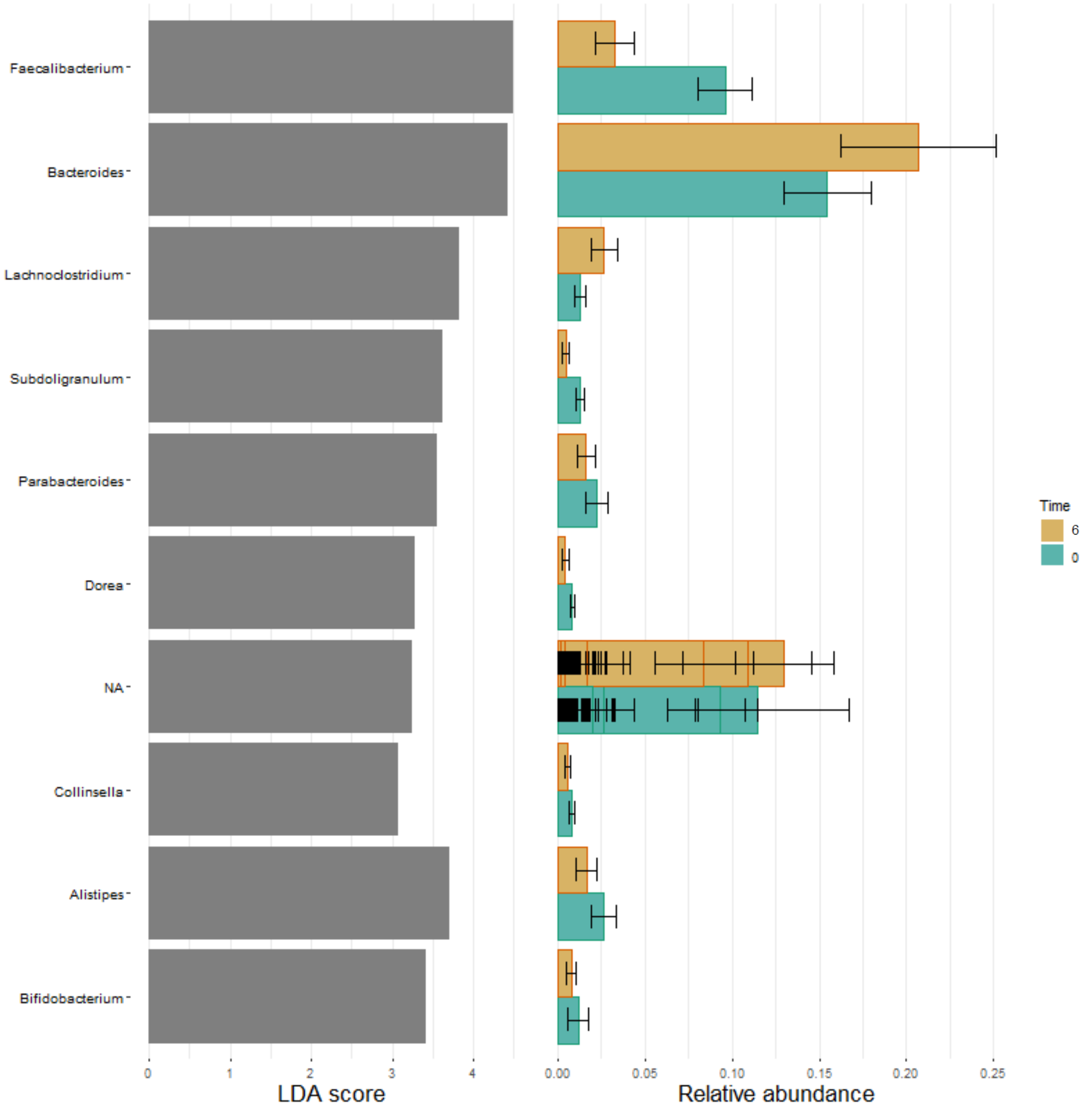

Supplement: Supplementary file 1 [file nutrients-14-00304-s001.zip › Figure S3.pdf]
