# Supplementary material for: The Effects of One Anastomosis Gastric Bypass Surgery on the Gastrointestinal Tract
Source: Nutrients. 2022 Jan 12;14(2):304. doi: 10.3390/nu14020304 (PMC8778673; doi:10.3390/nu14020304)
Supplement: Supplementary file 1 [file nutrients-14-00304-s001.zip › Figure S4.pdf]

Figure S4: Differeneces in alpha diversity (using the Shnannon Index)

A: Between the group who did not develop SIBO at 6 months post-surgery (SIBO negative, n=17) and the group who develpoed SIBO at 6 months post-surgery (SIBO positive, n=10) at baseline (A1, Time 0) and 6 months post-surgery (A2, Time 6)

B: Within the group who did not develop SIBO at 6 months post-surgery (B1, n=17) and within the group who developed SIBO at 6 months post-surgery (B2, n=10)

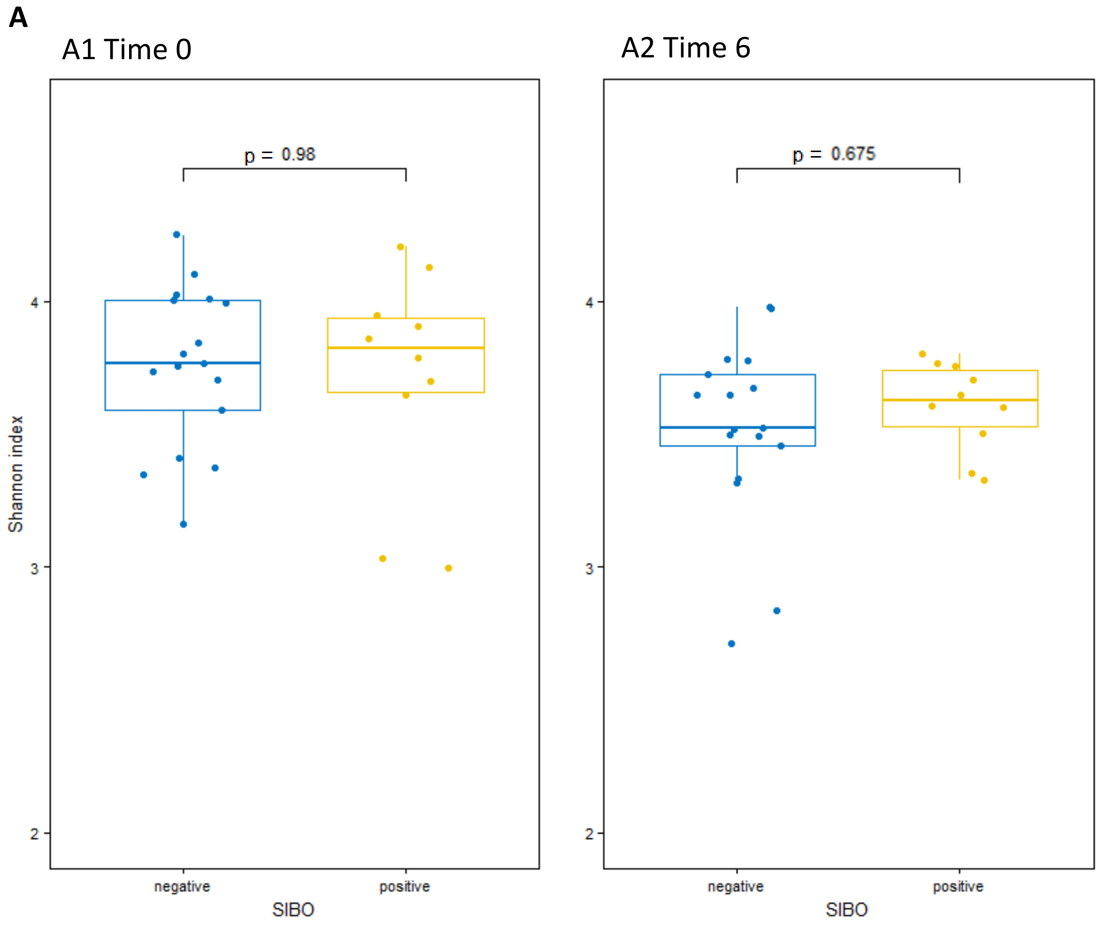

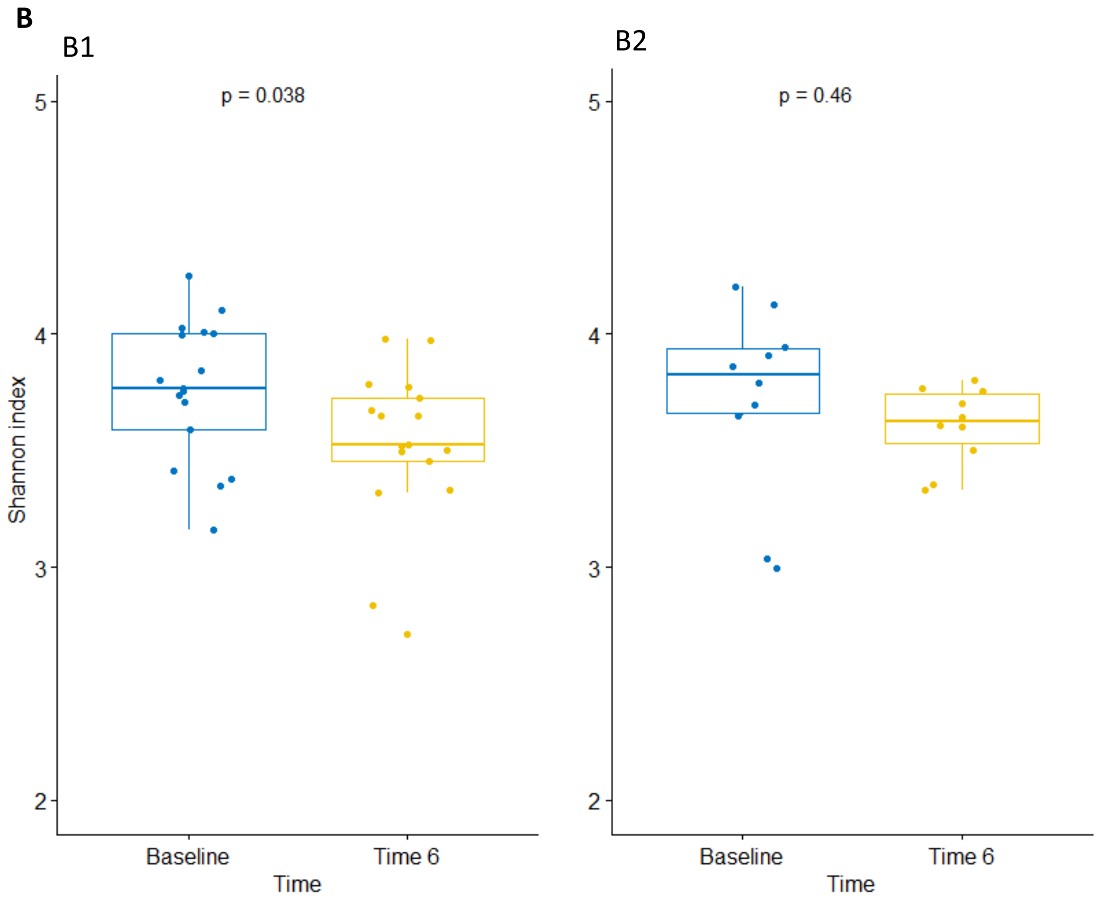
B
B1
B2
p = 0.038
p = 0.46
Shannon index
5
4
3
2
Baseline
Time 6
Time
