# Supplementary material for: The Effects of One Anastomosis Gastric Bypass Surgery on the Gastrointestinal Tract
Source: Nutrients. 2022 Jan 12;14(2):304. doi: 10.3390/nu14020304 (PMC8778673; doi:10.3390/nu14020304)

Figure S6: Differences in the delta of change of beta diversity (using the Unweighted Unifrac metric) over time between the group who did not develop SIBO at 6 months post-surgery (no SIBO at T6, n=17) and the group who developed SIBO at 6 months post-surgery (SIBO at T6, n=10)

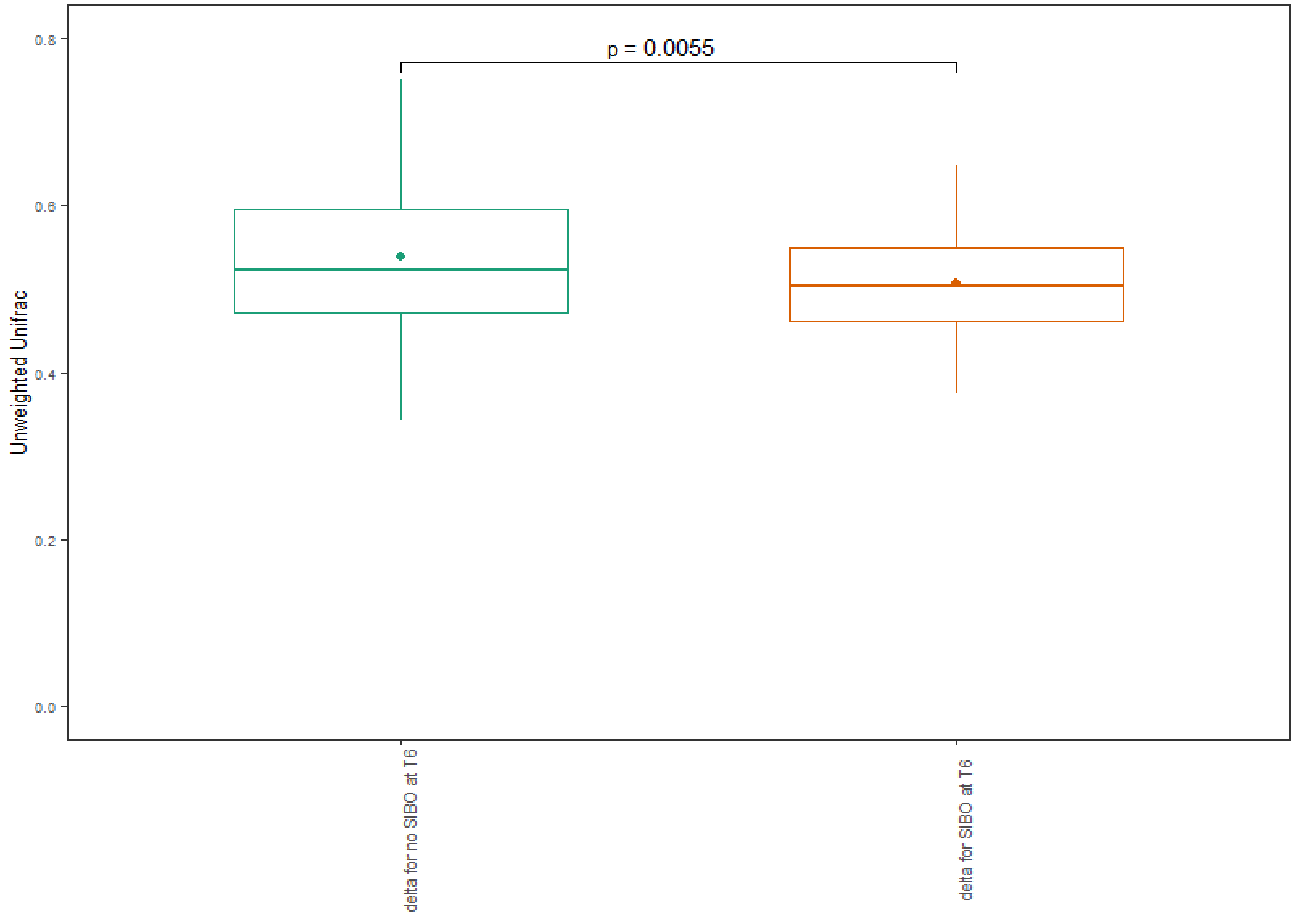

Supplement: Supplementary file 1 [file nutrients-14-00304-s001.zip › Figure S6.pdf]
