# Supplementary material for: The Effects of One Anastomosis Gastric Bypass Surgery on the Gastrointestinal Tract
Source: Nutrients. 2022 Jan 12;14(2):304. doi: 10.3390/nu14020304 (PMC8778673; doi:10.3390/nu14020304)

Figure S7: Differences in beta diversity (using the Unweighted Unifrac metric) when comparing samples according to FE1 test at 6 months post-surgery

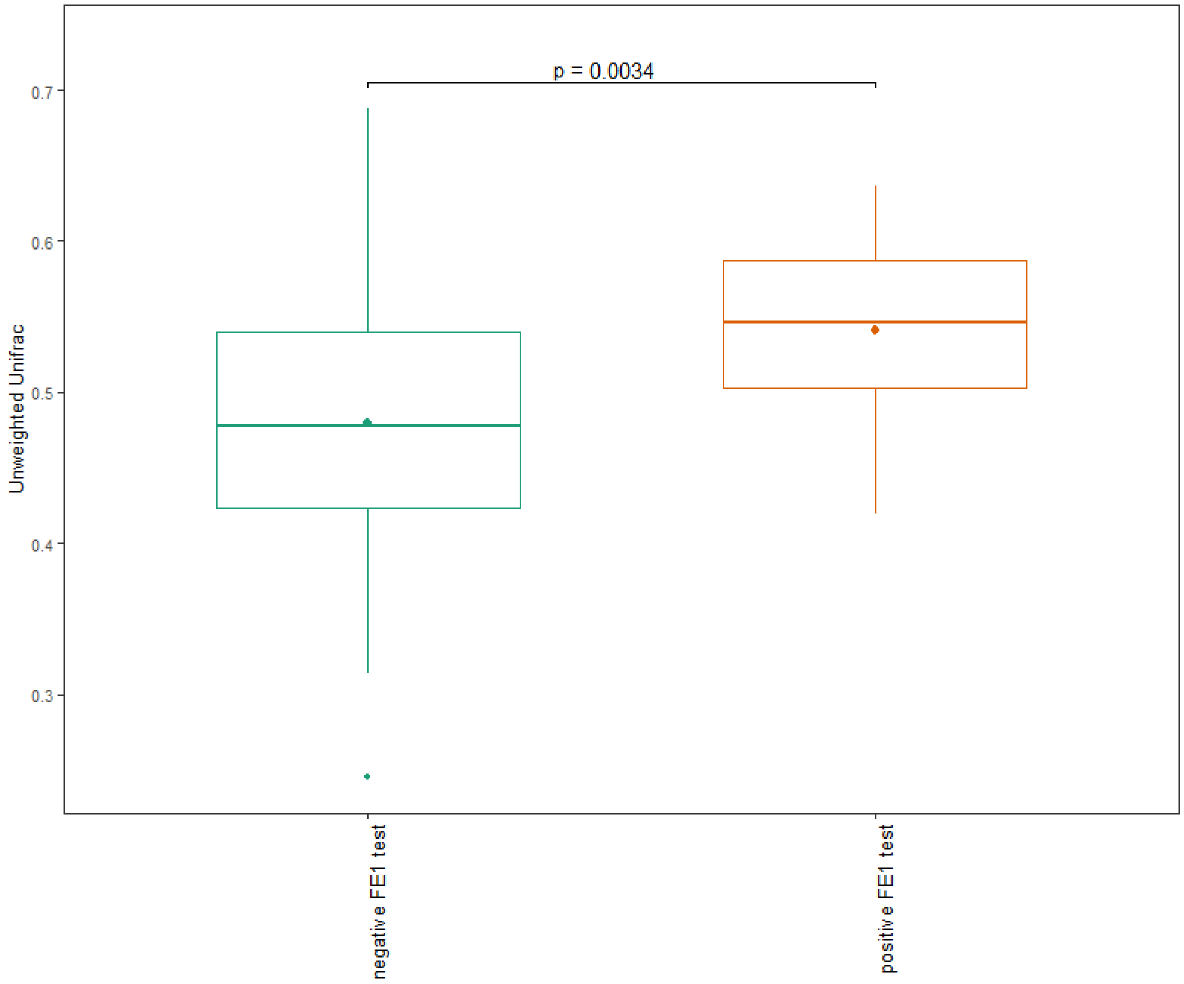

Supplement: Supplementary file 1 [file nutrients-14-00304-s001.zip › Figure S7.pdf]
