# Supplementary material for: The Effects of One Anastomosis Gastric Bypass Surgery on the Gastrointestinal Tract
Source: Nutrients. 2022 Jan 12;14(2):304. doi: 10.3390/nu14020304 (PMC8778673; doi:10.3390/nu14020304)
Supplement: Supplementary file 1 [file nutrients-14-00304-s001.zip › Methods S1.pdf]

Methods S1: Supplementary Methods

Pancreatic exocrine insufficiency assessment

The initial processing of the stools was performed using ScheBo stool sample extraction system (ScheBo Biotech AG, Giessen, Germany). FE1 concentrations were measured using ScheBo Pancreatic Elastase 1 ELISA kit (ScheBo Biotech AG) [1]. The experiments were carried out per manufacturer's instructions and in duplicate. Optical density at the absorbance of 405 nm was measured using Multiskan FC Microplate Photometer (Thermo Fisher Scientific Inc., Waltham, MA, USA).

16S amplicon sequencing and microbiome analysis

DNA was extracted from swabs using the DNeasy PowerSoil kit (Qiagen, Hilden, Germany) and the V4 region of 16S rRNA was amplified using primers 515F and 807R and processed per Illumina's 16S protocol on a MiSeq instrument (Illumina, San Diego, CA, USA). Raw sequence data were imported into the QIIME2 package (v2019.10) [2] for analysis using the DADA2 pipeline with the SILVA DB [3] (v132 at 99%, trained on the 515F and 807R [V4] subregion) [2, 4]. Samples were rarefied at a sampling depth of 24,252 using the diversity plugin (diversity core-metrics-phylogenetic) [5]. The rarefied feature table, rooted tree, taxonomic assignmentnts and metadata were imported into R (version 4.0.2) using the package qiime2R (v0.99.34) for further analyses using the R package microeco (v0.2.0), which included comparisons of relative abundance at the phyla and genera levels, comparisons of alpha and beta diversity, and differential abundance analysis. Plots were also generated using the R package microeco, unless stated otherwise.

Microbiome statistical analysis

Relative abundances between phyla and genera for all patients were compared between pre- and post-surgery time points using the paired Wilcoxon two-sided test (with FDR adjustment) from the R package rstatix (v0.6.0), where p-values < 0.05 were considered significant. Box plots were generated using the R packages ggplot2 (v3.3.2) and ggpubr (v0.4.0).

Alpha diversity measures were calculated for all samples, using a range of metrics (including Shannon index, Simpson index and Faith PD). Beta diversity measures were calculated using the metrics Bray-Curtis, Weighted and Unweighted UniFrac. For group comparisons in both alpha and beta diversity, either the Wilcoxon rank sum test (for dependent samples comparison) or Welch's t test (for independent samples comparison) were applied as appropriate. All p-values were FDR adjusted, and were considered significant when <0.05.

Differential abundance at the genus level was conducted using the Linear discriminant analysis effect size (LefSe) method [6] for (1) all patients between pre- and post-surgery time points, (2) patients who did not develop SIBO at 6 months post-surgery, (3) patients who developed SIBO at 6 months post-surgery.

References:

1. Tóth AZ, Szabó A, Hegyi E, Hegyi P, Sahin-Tóth M: **Detection of human elastase isoforms by the ScheBo Pancreatic Elastase 1 Test.** *Am J Physiol Gastrointest Liver Physiol* 2017, **312:**G606-G614.
2. Bolyen E, Rideout JR, Dillon MR, Bokulich NA, Abnet CC, Al-Ghalith GA, Alexander H, Alm EJ, Arumugam M, Asnicar F, et al: **Reproducible, interactive, scalable and extensible microbiome data science using QIIME 2.** *Nat Biotechnol* 2019, **37:**852-857.
3. Quast C, Pruesse E, Yilmaz P, Gerken J, Schweer T, Yarza P, Peplies J, Glöckner FO: **The SILVA ribosomal RNA gene database project: improved data processing and web-based tools.** *Nucleic Acids Res* 2013, **41:**D590-596.
4. Bokulich NA, Kaehler BD, Rideout JR, Dillon M, Bolyen E, Knight R, Huttley GA, Gregory Caporaso J: **Optimizing taxonomic classification of marker-gene amplicon sequences with QIIME 2's q2-feature-classifier plugin.** *Microbiome* 2018, **6:**90.
5. Weiss S, Xu ZZ, Peddada S, Amir A, Bittinger K, Gonzalez A, Lozupone C, Zaneveld JR, Vázquez-Baeza Y, Birmingham A, et al: **Normalization and microbial differential abundance strategies depend upon data characteristics.** *Microbiome* 2017, **5:**27.
6. Segata N, Izard J, Waldron L, Gevers D, Miropolsky L, Garrett WS, Huttenhower C: **Metagenomic biomarker discovery and explanation.** *Genome Biol* 2011, **12:**R60.
