# Supplementary material for: The Effects of One Anastomosis Gastric Bypass Surgery on the Gastrointestinal Tract
Source: Nutrients. 2022 Jan 12;14(2):304. doi: 10.3390/nu14020304 (PMC8778673; doi:10.3390/nu14020304)
Supplement: Supplementary file 1 [file nutrients-14-00304-s001.zip › Table S1.pdf]

Table S1: Changes in relative abundances of genera for all patients from baseline to 6 months post-surgery (n=28).

Only significant results (p<0.05) are listed.

Phyla	Genera	relative abundance at baseline (%)			relative abundance at 6 months post-surgery (%)			p-value (FDR adjusted)
		mean	min	max	mean	min	max	
Actinobacteria	Actinomyces	0.016	0	0.086	0.064	0	0.458	0.0229
Actinobacteria	Atopobium	0	0	0	0.016	0	0.111	0.0463
Actinobacteria	Rothia	0.002	0	0.061	0.029	0	0.181	0.0045
Bacteroidetes	Alistipes	3.659	0.037	16.75	1.978	0	9.153	0.0343
Bacteroidetes	Butyricimonas	0.044	0	0.334	0.130	0	0.536	0.0262
Bacteroidetes	gut metagenome	0.053	0	2.227	0.007	0	0.582	0.0192
Bacteroidetes	Prevotella 7	0.377	0	3.760	2.028	0	12.70	0.0054
Bacteroidetes	Vibrionimonas	0.032	0	0.136	0.004	0	0.070	0.0268
Firmicutes	Agathobacter	1.405	0	8.552	0.466	0	2.671	0.0088
Firmicutes	Anaerostipes	0.703	0	2.605	0.092	0	0.894	0.0009
Firmicutes	Blautia	3.561	0.346	14.06	0.191	0	1.015	<0.0001
Firmicutes	CAG-56	0.186	0	1.327	0.037	0	0.243	0.0343
Firmicutes	Clostridium sensu stricto 1	0.970	0	5.385	0.420	0	4.778	0.0234
Firmicutes	Coprococcus 1	0.072	0	0.254	0.028	0	0.173	0.0229
Firmicutes	Coprococcus 3	0.431	0.020	1.406	0.249	0	1.212	0.0200
Firmicutes	Dorea	1.268	0.160	5.096	0.430	0.028	2.961	0.0006
Firmicutes	Erysipelotrichaceae UCG-003	0.300	0	2.395	0.023	0	0.148	0.0017
Firmicutes	Eubacterium coprostanoligenes group	3.001	0	12.93	1.049	0	4.250	0.0261
Firmicutes	Eubacterium hallii group	0.836	0.024	5.908	0.033	0	0.311	<0.0001
Firmicutes	Fusicatenibacter	1.182	0.061	8.020	0.138	0	0.490	<0.0001
Firmicutes	Gemella	0.001	0	0.028	0.105	0	0.491	0.0009
Firmicutes	Holdemanella	0.631	0	3.138	0.122	0	1.142	0.0139
Firmicutes	Intestinibacter	0.219	0	1.666	0.008	0	0.206	0.0043
Firmicutes	Lachnoclostridium	1.159	0.065	5.533	2.368	0.243	11.59	0.0412
Firmicutes	Lachnospira	0.506	0	2.663	0.239	0	1.702	0.0458
Firmicutes	Lachnospiraceae FCS020 group	0.077	0	0.461	0.004	0	0.032	0.0003
Firmicutes	Lachnospiraceae ND3007 group	0.323	0	1.063	0.026	0	0.202	0.0002
Firmicutes	Lachnospiraceae UCG-004	0.101	0	0.400	1.565	0	17.87	0.0013
Firmicutes	Lachnospiraceae UCG-010	0.126	0	0.709	0.574	0	3.058	0.0229
Firmicutes	Marvinbryantia	0.046	0	0.413	0	0	0	0.0343

Phyla	Genera	relative abundance at baseline (%)			relative abundance at 6 months post-surgery (%)			p-value (FDR adjusted)
		**mean**	**min**	**max**	**mean**	**min**	**max**	
Firmicutes	Phascolarctobacterium	0.950	0	8.061	1.496	0	6.300	0.0394
Firmicutes	Romboutsia	1.350	0	23.88	0.059	0	0.961	0.0003
Firmicutes	Ruminiclostridium 5	0.168	0	0.940	0.052	0	0.210	0.0060
Firmicutes	Ruminococcaceae NK4A214 group	0.466	0	2.156	1.307	0	4.597	0.0394
Firmicutes	Ruminococcaceae UCG-003	0.286	0	1.778	1.685	0	6.553	0.0023
Firmicutes	Ruminococcaceae UCG-014	0.921	0	7.570	0.027	0	0.276	0.0035
Firmicutes	Ruminococcus gauvreauii group	0.261	0	1.789	0.003	0	0.070	0.0003
Firmicutes	Ruminococcus torques group	1.008	0.098	7.760	0.302	0	1.916	0.0004
Firmicutes	Streptococcus	0.358	0	2.779	2.486	0.016	8.154	<0.0001
Firmicutes	Subdoligranulum	1.561	0.185	3.661	0.631	0.028	4.202	0.0007
Firmicutes	Turicibacter	0.047	0	0.350	0.003	0	0.070	0.0458
Firmicutes	UBA1819	0.046	0	0.338	0.007	0	0.078	0.0331
Firmicutes	Veillonella	0.113	0	1.154	1.954	0	5.521	0.0002
Fusobacteria	Fusobacterium	0.320	0	8.910	0.861	0	15.25	0.0051
Proteobacteria	Escherichia-Shigella	0.848	0	7.793	6.383	0	27.87	0.0024
Proteobacteria	Neisseria	0	0	0	0.021	0	0.186	0.0343
Verrucomicrobia	Akkermansia	0.446	0	4.659	3.326	0	14.07	0.0030
