# Supplementary material for: The Effects of One Anastomosis Gastric Bypass Surgery on the Gastrointestinal Tract
Source: Nutrients. 2022 Jan 12;14(2):304. doi: 10.3390/nu14020304 (PMC8778673; doi:10.3390/nu14020304)
Supplement: Supplementary file 1 [file nutrients-14-00304-s001.zip › Table S2.pdf]

Table S2: Differential abundance analysis at the genera level using LefSe for all patients from baseline to 6 months post-surgery (n=28).

Only significant results ($p<0.05$) are listed.

The Time column indicates whether at baseline (Time 0) or at 6 months post-surgery (Time 6) the greater abundance was observed.

Phyla	Genera	Time	LDA	p-value (FDR)
Actinobacteria	Actinomyces	6	2.3991	<0.0001
Actinobacteria	Alloscardovia	0	1.9145	0.01599
Actinobacteria	Atopobium	0	2.1262	0.01083
Actinobacteria	Bifidobacterium	0	3.8399	<0.0001
Actinobacteria	Collinsella	0	3.179	<0.0001
Actinobacteria	Enterorhabdus	0	1.5364	0.00353
Actinobacteria	Libanicoccus	0	-1.328	0.02401
Actinobacteria	Rothia	6	2.1755	<0.0001
Actinobacteria	Senegalimassilia	0	1.6461	<0.0001
Actinobacteria	Slackia	6	1.1454	<0.0001
Bacteroidetes	Alistipes	0	3.9193	<0.0001
Bacteroidetes	Alloprevotella	0	3.4543	0.01083
Bacteroidetes	Bacteroides	6	3.9585	<0.0001
Bacteroidetes	Barnesiella	0	3.3749	<0.0001
Bacteroidetes	Butyricimonas	6	2.6375	<0.0001
Bacteroidetes	Coprobacter	0	1.7102	<0.0001
Bacteroidetes	metagenome	0	3.1934	0.01083
Bacteroidetes	Odoribacter	0	2.793	<0.0001
Bacteroidetes	Parabacteroides	0	3.4325	<0.0001
Bacteroidetes	Paraprevotella	0	3.1172	<0.0001
Bacteroidetes	Porphyromonas	0	1.6627	0.02401
Bacteroidetes	Prevotella	0	1.9188	0.00512
Bacteroidetes	Prevotella 6	0	2.1995	0.01599
Bacteroidetes	Prevotella 7	6	3.9184	<0.0001
Bacteroidetes	Prevotella 9	6	3.9982	<0.0001
Bacteroidetes	Rikenellaceae RC9 gut group	0	2.7719	0.00512
Bacteroidetes	uncultured bacterium	0	2.6553	0.00736
Bacteroidetes	Vibrionimonas	0	2.1295	<0.0001
Epsilonbacteraeota	Campylobacter	0	1.0916	0.00512
Euryarchaeota	Methanobrevibacter	0	1.9312	0.00163
Firmicutes	[Eubacterium] brachy group	0	2.0683	0.03603
Firmicutes	[Eubacterium] coprostanoligenes group	0	3.9922	<0.0001
Firmicutes	[Eubacterium] eligens group	0	2.9274	<0.0001
Firmicutes	[Eubacterium] hallii group	0	3.598	<0.0001
Firmicutes	[Eubacterium] ruminantium group	0	2.6033	0.00012

Phyla	Genera	Time	LDA	p-value (FDR)
Firmicutes	[Eubacterium] ventriosum group	0	2.8216	<0.0001
Firmicutes	[Eubacterium] xylanophilum group	0	2.4257	0.00012
Firmicutes	[Ruminococcus] gauvreauii group	0	3.109	<0.0001
Firmicutes	[Ruminococcus] gnavus group	0	2.4608	0.00163
Firmicutes	[Ruminococcus] torques group	0	3.5373	<0.0001
Firmicutes	Acidaminococcus	6	3.6251	<0.0001
Firmicutes	Agathobacter	0	3.6753	<0.0001
Firmicutes	Allisonella	6	2.871	<0.0001
Firmicutes	Anaerosporobacter	0	2.0268	0.00512
Firmicutes	Anaerostipes	0	3.4802	<0.0001
Firmicutes	Angelakisella	0	1.3635	0.02401
Firmicutes	Blautia	0	4.223	<0.0001
Firmicutes	Butyricicoccus	0	2.7542	<0.0001
Firmicutes	CAG-56	0	2.8771	<0.0001
Firmicutes	Catenibacterium	6	3.3667	<0.0001
Firmicutes	Christensenellaceae R-7 group	0	3.2818	<0.0001
Firmicutes	Clostridium sensu stricto 1	0	3.4412	<0.0001
Firmicutes	Coprococcus 1	0	2.2722	<0.0001
Firmicutes	Coprococcus 2	0	3.1215	<0.0001
Firmicutes	Coprococcus 3	0	2.9568	<0.0001
Firmicutes	Defluviitaleaceae UCG-011	0	1.1877	0.02401
Firmicutes	Dialister	0	3.3761	<0.0001
Firmicutes	Dorea	0	3.6159	<0.0001
Firmicutes	DTU089	0	1.2782	0.03603
Firmicutes	Erysipelatoclostridium	0	1.6167	0.00736
Firmicutes	Erysipelotrichaceae UCG-003	0	3.1359	<0.0001
Firmicutes	Faecalibacterium	0	4.1741	<0.0001
Firmicutes	Family XIII AD3011 group	0	1.9533	<0.0001
Firmicutes	Family XIII UCG-001	0	1.4625	0.00055
Firmicutes	Flavonifractor	0	2.079	<0.0001
Firmicutes	Fusicatenibacter	0	3.7119	<0.0001
Firmicutes	GCA-900066575	0	2.0277	<0.0001
Firmicutes	Gemella	6	2.7385	<0.0001
Firmicutes	Granulicatella	0	2.3649	0.00353
Firmicutes	Holdemanella	0	3.409	<0.0001
Firmicutes	Holdemania	0	-1.4695	0.01599
Firmicutes	Howardella	0	0.9557	<0.0001
Firmicutes	Hungatella	0	2.3804	0.00736
Firmicutes	Intestinibacter	0	3.0218	0.00026
Firmicutes	Intestinimonas	0	2.4784	0.00163
Firmicutes	Lachnoanaerobaculum	0	2.1704	0.03603
Firmicutes	Lachnoclostridium	6	3.7736	<0.0001

Phyla	Genera	Time	LDA	p-value (FDR)
Firmicutes	Lachnospira	0	3.132	<0.0001
Firmicutes	Lachnospiraceae FCS020 group	0	2.5365	<0.0001
Firmicutes	Lachnospiraceae ND3007 group	0	3.1674	<0.0001
Firmicutes	Lachnospiraceae NK4A136 group	0	3.2439	<0.0001
Firmicutes	Lachnospiraceae UCG-001	6	2.9101	<0.0001
Firmicutes	Lachnospiraceae UCG-004	6	3.8651	<0.0001
Firmicutes	Lachnospiraceae UCG-008	6	2.4129	<0.0001
Firmicutes	Lachnospiraceae UCG-010	6	3.3581	<0.0001
Firmicutes	Lactobacillus	0	3.4031	<0.0001
Firmicutes	Lactococcus	0	1.5068	0.01599
Firmicutes	Marvinbryantia	0	2.2898	0.00736
Firmicutes	Megamonas	0	3.5914	0.0008
Firmicutes	Megasphaera	6	3.5745	<0.0001
Firmicutes	Mitsuokella	0	2.5586	0.01083
Firmicutes	Mogibacterium	0	1.6028	0.00512
Firmicutes	Moryella	0	2.0105	0.00736
Firmicutes	NA	6	3.4413	<0.0001
Firmicutes	NA	0	2.7646	<0.0001
Firmicutes	NA	0	2.4214	0.01599
Firmicutes	NA	0	1.8531	0.03603
Firmicutes	Negativibacillus	0	2.5391	0.00055
Firmicutes	Oscillibacter	0	2.2587	<0.0001
Firmicutes	Oscillospira	0	1.767	0.01083
Firmicutes	Peptococcus	0	2.0554	0.01599
Firmicutes	Phascolarctobacterium	6	3.4351	<0.0001
Firmicutes	Romboutsia	0	3.8056	<0.0001
Firmicutes	Roseburia	6	3.4837	<0.0001
Firmicutes	Ruminiclostridium 5	0	2.7523	<0.0001
Firmicutes	Ruminiclostridium 6	0	2.8928	0.00117
Firmicutes	Ruminiclostridium 9	6	3.0586	<0.0001
Firmicutes	Ruminococcaceae NK4A214 group	6	3.622	<0.0001
Firmicutes	Ruminococcaceae UCG-002	6	3.6086	<0.0001
Firmicutes	Ruminococcaceae UCG-003	6	3.8454	<0.0001
Firmicutes	Ruminococcaceae UCG-005	0	2.7916	<0.0001
Firmicutes	Ruminococcaceae UCG-010	0	2.5008	<0.0001
Firmicutes	Ruminococcaceae UCG-013	0	2.581	<0.0001
Firmicutes	Ruminococcaceae UCG-014	0	3.6531	<0.0001
Firmicutes	Ruminococcus 1	6	3.2331	<0.0001
Firmicutes	Ruminococcus 2	0	3.7428	<0.0001
Firmicutes	Solobacterium	0	1.364	0.01083
Firmicutes	Streptococcus	6	4.0252	<0.0001
Firmicutes	Subdoligranulum	0	3.6657	<0.0001
Firmicutes	Terrisporobacter	0	2.7294	0.02401

Phyla	Genera	Time	LDA	p-value (FDR)
Firmicutes	Turicibacter	0	2.3314	0.00353
Firmicutes	Tyzzerella 3	0	2.1017	0.02401
Firmicutes	UBA1819	0	2.2168	<0.0001
Firmicutes	Veillonella	6	3.9624	<0.0001
Firmicutes	Weissella	0	2.4641	0.00353
Fusobacteria	Fusobacterium	6	3.4936	<0.0001
Fusobacteria	Leptotrichia	0	2.0605	0.01599
NA	NA	0	1.7528	0.00163
Patescibacteria	NA	0	1.888	0.00353
Proteobacteria	Aggregatibacter	0	2.2151	0.01599
Proteobacteria	Bilophila	6	2.8638	<0.0001
Proteobacteria	Desulfovibrio	0	3.1768	0.00018
Proteobacteria	Escherichia-Shigella	6	4.4425	<0.0001
Proteobacteria	gut metagenome	0	1.8146	0.03603
Proteobacteria	Haemophilus	6	3.2947	<0.0001
Proteobacteria	Mailhella	0	2.0312	0.01083
Proteobacteria	NA	6	4.3083	<0.0001
Proteobacteria	NA	0	2.2146	0.01599
Proteobacteria	Neisseria	0	2.2127	0.00736
Proteobacteria	Oxalobacter	0	-1.4287	0.00736
Proteobacteria	Parasutterella	0	2.5246	<0.0001
Proteobacteria	Pseudomonas	0	3.5587	0.00038
Proteobacteria	Sutterella	6	3.3623	<0.0001
Proteobacteria	Undibacterium	0	1.6904	0.02401
Tenericutes	gut metagenome	0	3.0932	0.00736
Verrucomicrobia	Akkermansia	6	4.1584	<0.0001
