# Supplementary material for: The Effects of One Anastomosis Gastric Bypass Surgery on the Gastrointestinal Tract
Source: Nutrients. 2022 Jan 12;14(2):304. doi: 10.3390/nu14020304 (PMC8778673; doi:10.3390/nu14020304)
Supplement: Supplementary file 1 [file nutrients-14-00304-s001.zip › Table S3.pdf]

Table S3: Differential abundance analysis at the genera level using LefSe for patients who did not develop SIBO from baseline to 6 months post-surgery (n=17).

Only significant results (p<0.05) are listed.

The Time column indicates whether at baseline (Time 0) or at 6 months post-surgery (Time 6) the greater abundance was observed.

**Phyla**	**Genera**	**Time**	**LDA**	**p-value (FDR)**
Actinobacteria	Actinomyces	6	2.47437	0.000615
Actinobacteria	Atopobium	0	2.33642	0.036032
Actinobacteria	Bifidobacterium	0	3.99126	<0.0001
Actinobacteria	Collinsella	0	3.30426	<0.0001
Actinobacteria	Rothia	6	2.54022	0.001744
Actinobacteria	Senegalimassilia	6	1.91949	0.000615
Actinobacteria	Slackia	0	1.65852	0.000615
Bacteroidetes	Alistipes	0	3.98399	<0.0001
Bacteroidetes	Bacteroides	0	4.26798	<0.0001
Bacteroidetes	Barnesiella	0	3.40612	<0.0001
Bacteroidetes	Butyricimonas	6	2.48608	0.000314
Bacteroidetes	Coprobacter	0	2.08501	0.002489
Bacteroidetes	metagenome	0	3.40450	0.036032
Bacteroidetes	Odoribacter	6	2.98106	<0.0001
Bacteroidetes	Parabacteroides	0	3.43841	<0.0001
Bacteroidetes	Paraprevotella	0	2.68792	0.003566
Bacteroidetes	Prevotella	0	2.03500	0.010811
Bacteroidetes	Prevotella 7	6	3.94954	0.001189
Bacteroidetes	Prevotella 9	0	4.09395	0.000152
Bacteroidetes	Vibrionimonas	0	2.22826	0.002489
Epsilonbacteraeota	Campylobacter	0	2.28070	0.036032
Euryarchaeota	Methanobrevibacter	0	2.11131	0.005078
Firmicutes	[Eubacterium] coprostanoligenes group	0	3.96243	<0.0001
Firmicutes	[Eubacterium] eligens group	0	2.92700	<0.0001
Firmicutes	[Eubacterium] hallii group	0	3.67962	0.000110
Firmicutes	[Eubacterium] ruminantium group	0	2.89918	0.005078
Firmicutes	[Eubacterium] ventriosum group	0	2.64212	<0.0001
Firmicutes	[Eubacterium] xylanophilum group	0	2.69648	0.002489
Firmicutes	[Ruminococcus] gauvreauii group	0	3.05279	0.001189
Firmicutes	[Ruminococcus] gnavus group	0	2.60604	0.024637
Firmicutes	[Ruminococcus] torques group	0	3.65710	<0.0001
Firmicutes	Acidaminococcus	6	3.60721	0.000443
Firmicutes	Agathobacter	0	3.65082	<0.0001
Firmicutes	Allisonella	6	2.85323	0.000443

**Phyla**	**Genera**	**Time**	**LDA**	**p-value (FDR)**
Firmicutes	Anaerosporobacter	0	2.02288	0.010811
Firmicutes	Anaerostipes	0	3.57361	<0.0001
Firmicutes	Blautia	0	4.25711	<0.0001
Firmicutes	Butyricicoccus	0	2.36595	<0.0001
Firmicutes	CAG-56	0	2.50428	0.000152
Firmicutes	Catenibacterium	6	3.38684	0.000873
Firmicutes	Christensenellaceae R-7 group	0	3.59683	<0.0001
Firmicutes	Clostridium sensu stricto 1	0	3.33491	<0.0001
Firmicutes	Coprococcus 1	0	1.86148	<0.0001
Firmicutes	Coprococcus 2	0	3.02589	<0.0001
Firmicutes	Coprococcus 3	0	2.94328	<0.0001
Firmicutes	Dialister	0	3.50317	0.000615
Firmicutes	Dorea	0	3.69078	<0.0001
Firmicutes	Erysipelatoclostridium	0	1.17173	0.036032
Firmicutes	Erysipelotrichaceae UCG-003	0	3.06643	<0.0001
Firmicutes	Faecalibacterium	0	3.83756	<0.0001
Firmicutes	Family XIII AD3011 group	6	2.32949	0.000110
Firmicutes	Family XIII UCG-001	0	1.94142	0.007591
Firmicutes	Flavonifractor	0	2.27456	0.000873
Firmicutes	Fusicatenibacter	0	3.80371	<0.0001
Firmicutes	GCA-900066575	0	2.36131	0.001189
Firmicutes	Gemella	6	2.84506	0.001189
Firmicutes	Holdemanella	0	3.41496	0.000220
Firmicutes	Howardella	0	1.96292	0.001744
Firmicutes	Intestinibacter	0	2.99657	0.002489
Firmicutes	Intestinimonas	0	2.60841	0.005078
Firmicutes	Lachnoclostridium	6	3.74344	<0.0001
Firmicutes	Lachnospira	0	3.29613	<0.0001
Firmicutes	Lachnospiraceae FCS020 group	0	2.56196	0.000314
Firmicutes	Lachnospiraceae ND3007 group	0	3.14081	<0.0001
Firmicutes	Lachnospiraceae NK4A136 group	0	2.80055	<0.0001
Firmicutes	Lachnospiraceae UCG-001	6	2.77068	0.000873
Firmicutes	Lachnospiraceae UCG-004	6	3.92015	<0.0001
Firmicutes	Lachnospiraceae UCG-008	6	2.66510	0.000443
Firmicutes	Lachnospiraceae UCG-010	6	3.52177	<0.0001
Firmicutes	Lactobacillus	0	2.55382	0.001189
Firmicutes	Megamonas	0	3.37938	0.016310
Firmicutes	Megasphaera	6	3.41380	0.000443
Firmicutes	Mogibacterium	0	2.19159	0.036032
Firmicutes	Moryella	0	2.07807	0.024637
Firmicutes	NA	6	3.55901	<0.0001
Firmicutes	NA	0	2.70070	0.003566

Phyla	Genera	Time	LDA	p-value (FDR)
Firmicutes	Negativibacillus	0	2.54834	0.010811
Firmicutes	Oscillibacter	0	2.16643	<0.0001
Firmicutes	Phascolarctobacterium	6	3.53791	<0.0001
Firmicutes	Romboutsia	0	3.95153	0.000220
Firmicutes	Roseburia	6	3.70589	<0.0001
Firmicutes	Ruminiclostridium 5	0	2.71704	<0.0001
Firmicutes	Ruminiclostridium 6	0	3.16876	0.010811
Firmicutes	Ruminiclostridium 9	6	3.09830	<0.0001
Firmicutes	Ruminococcaceae NK4A214 group	6	3.70476	<0.0001
Firmicutes	Ruminococcaceae UCG-002	6	3.69112	<0.0001
Firmicutes	Ruminococcaceae UCG-003	6	3.91256	<0.0001
Firmicutes	Ruminococcaceae UCG-005	0	2.96053	0.000152
Firmicutes	Ruminococcaceae UCG-010	0	2.67039	0.003566
Firmicutes	Ruminococcaceae UCG-013	6	2.44063	<0.0001
Firmicutes	Ruminococcaceae UCG-014	0	3.79336	0.003566
Firmicutes	Ruminococcus 1	6	3.35095	<0.0001
Firmicutes	Ruminococcus 2	0	2.93209	0.000152
Firmicutes	Streptococcus	6	3.97091	<0.0001
Firmicutes	Subdoligranulum	0	3.69734	<0.0001
Firmicutes	Turicibacter	0	2.49577	0.005078
Firmicutes	UBA1819	0	2.21803	0.002489
Firmicutes	Veillonella	6	3.94845	<0.0001
Fusobacteria	Fusobacterium	6	3.65362	0.001189
NA	NA	0	1.78298	0.010811
Patescibacteria	NA	0	2.40167	0.010811
Proteobacteria	Aggregatibacter	0	2.42039	0.036032
Proteobacteria	Bilophila	6	2.79337	<0.0001
Proteobacteria	Desulfovibrio	0	3.43725	0.003566
Proteobacteria	Escherichia-Shigella	6	4.34237	<0.0001
Proteobacteria	Haemophilus	6	3.37125	<0.0001
Proteobacteria	Mailhella	0	2.43306	0.024637
Proteobacteria	NA	6	4.43556	<0.0001
Proteobacteria	Neisseria	0	2.34937	0.036032
Proteobacteria	Parasutterella	6	2.62098	0.000220
Proteobacteria	Pseudomonas	0	3.80916	0.003566
Proteobacteria	Sutterella	6	3.25034	0.000110
Tenericutes	gut metagenome	0	2.69962	0.036032
Verrucomicrobia	Akkermansia	6	4.23213	<0.0001
