# Supplementary material for: The Effects of One Anastomosis Gastric Bypass Surgery on the Gastrointestinal Tract
Source: Nutrients. 2022 Jan 12;14(2):304. doi: 10.3390/nu14020304 (PMC8778673; doi:10.3390/nu14020304)
Supplement: Supplementary file 1 [file nutrients-14-00304-s001.zip › Table S4.pdf]

Table S4: Differential abundance analysis at the genera level using LefSe for patients who developed SIBO from baseline to 6 months post-surgery (n=10).

Only the top 10 significant results (p<0.05) are presented.
The Time column indicates whether at baseline (Time 0) or at 6 months post-surgery (Time 6) the greater abundance was observed.

**Phyla**	**Genera**	**Time**	**LDA**	**p-value (FDR)**
Actinobacteria	Actinomyces	0	2.32398	0.006124
Actinobacteria	Bifidobacterium	0	3.41783	0.000831
Actinobacteria	Collinsella	0	3.08105	<0.0001
Actinobacteria	Enterorhabdus	0	-2.65778	0.024310
Actinobacteria	Rothia	6	1.73128	0.017231
Actinobacteria	Senegalimassilia	0	-1.79565	0.006124
Actinobacteria	Slackia	6	-1.94653	0.002431
Bacteroidetes	Alistipes	0	3.70886	0.000831
Bacteroidetes	Alloprevotella	0	3.66642	0.036032
Bacteroidetes	Bacteroides	6	4.42618	<0.0001
Bacteroidetes	Barnesiella	0	3.32290	0.001075
Bacteroidetes	Butyricimonas	6	2.84436	0.008106
Bacteroidetes	Coprobacter	0	2.39462	0.012046
Bacteroidetes	Odoribacter	0	2.62999	0.002431
Bacteroidetes	Parabacteroides	0	3.55874	<0.0001
Bacteroidetes	Paraprevotella	0	3.45324	0.006124
Bacteroidetes	Prevotella 7	6	3.93528	0.004524
Bacteroidetes	Prevotella 9	6	4.23887	0.001075
Bacteroidetes	Rikenellaceae RC9 gut group	0	3.15096	0.024310
Bacteroidetes	Vibrionimonas	0	-2.19035	0.008106
Firmicutes	[Eubacterium] coprostanoligenes group	0	4.03694	0.000929
Firmicutes	[Eubacterium] eligens group	6	3.09285	0.001075
Firmicutes	[Eubacterium] hallii group	0	3.42886	0.003323
Firmicutes	[Eubacterium] ruminantium group	6	3.07607	0.008106
Firmicutes	[Eubacterium] ventriosum group	0	2.33256	0.006124
Firmicutes	[Eubacterium] xylanophilum group	0	2.39404	0.024310
Firmicutes	[Ruminococcus] gauvreauii group	0	3.15585	0.008106
Firmicutes	[Ruminococcus] gnavus group	0	1.87021	0.024310
Firmicutes	[Ruminococcus] torques group	0	3.31038	0.001415
Firmicutes	Acidaminococcus	6	3.65443	0.008106
Firmicutes	Agathobacter	0	3.65412	0.002431
Firmicutes	Allisonella	6	2.95693	0.004524
Firmicutes	Anaerostipes	0	3.18590	0.003323
Firmicutes	Blautia	0	4.15585	0.000929
Firmicutes	Butyricicoccus	0	3.07595	0.006124

**Phyla**	**Genera**	**Time**	**LDA**	**p-value (FDR)**
Firmicutes	CAG-56	0	3.16711	0.006124
Firmicutes	Catenibacterium	0	2.90804	0.036032
Firmicutes	Christensenellaceae R-7 group	0	3.38231	0.001880
Firmicutes	Clostridium sensu stricto 1	0	3.61798	0.003323
Firmicutes	Coprococcus 1	0	2.10996	0.008106
Firmicutes	Coprococcus 2	0	3.39386	0.004524
Firmicutes	Coprococcus 3	0	2.97503	0.000831
Firmicutes	Dialister	0	3.73533	0.004524
Firmicutes	Dorea	0	3.28866	<0.0001
Firmicutes	Erysipelotrichaceae UCG-003	0	3.26947	0.006124
Firmicutes	Faecalibacterium	0	4.50167	<0.0001
Firmicutes	Family XIII AD3011 group	0	2.22818	0.036032
Firmicutes	Flavonifractor	0	-1.94033	0.008106
Firmicutes	Fusicatenibacter	0	3.58055	0.001415
Firmicutes	GCA-900066575	0	2.35999	0.012046
Firmicutes	Gemella	6	2.71243	0.008106
Firmicutes	Granulicatella	6	2.59802	0.017231
Firmicutes	Holdemanella	0	3.43778	0.003323
Firmicutes	Howardella	0	-2.48367	0.017231
Firmicutes	Lachnoclostridium	6	3.83441	<0.0001
Firmicutes	Lachnospira	0	2.81364	0.001415
Firmicutes	Lachnospiraceae FCS020 group	0	2.12789	0.008106
Firmicutes	Lachnospiraceae ND3007 group	0	3.20599	0.002431
Firmicutes	Lachnospiraceae NK4A136 group	0	3.59589	0.001415
Firmicutes	Lachnospiraceae UCG-001	6	3.08578	0.012046
Firmicutes	Lachnospiraceae UCG-004	6	3.74846	0.001415
Firmicutes	Lachnospiraceae UCG-008	0	-0.60424	0.024310
Firmicutes	Lachnospiraceae UCG-010	6	2.78017	0.004524
Firmicutes	Lactobacillus	0	3.86728	0.024310
Firmicutes	Megamonas	0	3.82966	0.024310
Firmicutes	Megasphaera	6	3.78541	0.012046
Firmicutes	Mitsuokella	0	2.93761	0.024310
Firmicutes	NA	6	3.25284	<0.0001
Firmicutes	NA	0	2.73324	0.008106
Firmicutes	Negativibacillus	0	1.16104	0.036032
Firmicutes	Oscillibacter	6	2.66064	0.001880
Firmicutes	Phascolarctobacterium	6	3.31999	0.003323
Firmicutes	Romboutsia	0	3.48484	0.008106
Firmicutes	Roseburia	0	3.30054	0.000831
Firmicutes	Ruminiclostridium 5	0	2.40957	0.003323
Firmicutes	Ruminiclostridium 9	0	3.02299	0.000929
Firmicutes	Ruminococcaceae NK4A214 group	6	3.46188	0.001880
Firmicutes	Ruminococcaceae UCG-002	6	3.79047	0.000929

Phyla	Genera	Time	LDA	p-value (FDR)
Firmicutes	Ruminococcaceae UCG-003	6	3.72802	0.001075
Firmicutes	Ruminococcaceae UCG-005	6	2.96344	0.002431
Firmicutes	Ruminococcaceae UCG-010	0	2.21373	0.008106
Firmicutes	Ruminococcaceae UCG-013	0	3.11744	0.000929
Firmicutes	Ruminococcaceae UCG-014	0	3.06026	0.006124
Firmicutes	Ruminococcus 1	6	3.38604	0.000929
Firmicutes	Ruminococcus 2	0	4.04750	0.001075
Firmicutes	Streptococcus	6	4.11044	0.000929
Firmicutes	Subdoligranulum	0	3.62469	<0.0001
Firmicutes	UBA1819	0	-2.50748	0.008106
Firmicutes	Veillonella	6	3.98980	0.001880
Firmicutes	Weissella	6	2.86476	0.017231
Fusobacteria	Fusobacterium	6	3.10062	0.006124
Proteobacteria	Bilophila	6	3.11374	0.001415
Proteobacteria	Desulfovibrio	0	3.20316	0.024310
Proteobacteria	Escherichia-Shigella	6	4.57254	0.001075
Proteobacteria	Haemophilus	6	3.91277	0.001415
Proteobacteria	NA	6	3.89509	0.004524
Proteobacteria	Oxalobacter	6	1.98112	0.024310
Proteobacteria	Parasutterella	0	2.76692	0.017231
Proteobacteria	Pseudomonas	0	2.86428	0.036032
Proteobacteria	Sutterella	6	3.52781	0.000929
Verrucomicrobia	Akkermansia	6	4.04741	0.002431
