# Supplementary material for: The Effects of One Anastomosis Gastric Bypass Surgery on the Gastrointestinal Tract
Source: Nutrients. 2022 Jan 12;14(2):304. doi: 10.3390/nu14020304 (PMC8778673; doi:10.3390/nu14020304)
Supplement: Supplementary file 1 [file nutrients-14-00304-s001.zip › Table S5.pdf]

Table S5: Comparison of the differential abundance analysis at the genera level using LefSe results for patients who did not develop SIBO from baseline to 6 months post-surgery (SIBO neg) and patients who did develop SIBO (SIBO pos).

Shared indicates genera that showed significant differences in both groups, with either positive or negative correlation.

SIBO neg-unique indicates genera that showed significant differences only in the SIBO neg group, while SIBO pos-unique indicates genera that showed significant differences only in the SIBO pos group.

Time indicates whether the greater abundance was at baseline (Time 0) or at 6 months post-surgery (Time 6).

Shared				SIBO neg-unique			SIBO pos-unique		
**Phyla**	**Genera**	**Correlation**	**Time**	**Phyla**	**Genera**	**Time**	**Phyla**	**Genera**	**Time**
Actinobacteria	Bifidobacterium	pos	0	Actinobacteria	Atopobium	0	Actinobacteria	Enterorhabdus	0
Actinobacteria	Collinsella	pos	0	Bacteroidetes	metagenome	0	Bacteroidetes	Alloprevotella	0
Actinobacteria	Rothia	pos	6	Bacteroidetes	Prevotella	0	Bacteroidetes	Rikenellaceae RC9 gut group	0
Bacteroidetes	Alistipes	pos	0	Epsilonbacteraeota	Campylobacter	0	Firmicutes	Granulicatella	6
Bacteroidetes	Barnesiella	pos	0	Euryarchaeota	Methanobrevibacter	0	Firmicutes	Mitsuokella	0
Bacteroidetes	Butyricimonas	pos	6	Firmicutes	Anaerosporobacter	0	Firmicutes	Weissella	6
Bacteroidetes	Coprobacter	pos	0	Firmicutes	Erysipelatoclostridium	0	Proteobacteria	Oxalobacter	6
Bacteroidetes	Parabacteroides	pos	0	Firmicutes	Family XIII UCG-001	0			
Bacteroidetes	Paraprevotella	pos	0	Firmicutes	Intestinibacter	0			
Bacteroidetes	Prevotella 7	pos	6	Firmicutes	Intestinimonas	0			
Bacteroidetes	Vibrionimonas	pos	0	Firmicutes	Mogibacterium	0			
Firmicutes	[Eubacterium] coprostanoligenes group	pos	0	Firmicutes	Moryella	0			
Firmicutes	[Eubacterium] hallii group	pos	0	Firmicutes	Ruminiclostridium 6	0			

Shared				SIBO neg-unique			SIBO pos-unique		
Firmicutes	[Eubacterium] ventriosum group	pos	0	Firmicutes	Turicibacter	0			
Firmicutes	[Eubacterium] xylanophilum group	pos	0	Proteobacteria	Aggregatibacter	0			
Firmicutes	[Ruminococcus] gauvreauii group	pos	0	Proteobacteria	Mailhella	0			
Firmicutes	[Ruminococcus] gnavus group	pos	0	Proteobacteria	Neisseria	0			
Firmicutes	[Ruminococcus] torques group	pos	0	Tenericutes	gut metagenome	0			
Firmicutes	Acidaminococcus	pos	6						
Firmicutes	Agathobacter	pos	0						
Firmicutes	Allisonella	pos	6						
Firmicutes	Anaerostipes	pos	0						
Firmicutes	Blautia	pos	0						
Firmicutes	Butyricicoccus	pos	0						
Firmicutes	CAG-56	pos	0						
Firmicutes	Christensenellaceae R-7 group	pos	0						
Firmicutes	Clostridium sensu stricto 1	pos	0						
Firmicutes	Coprococcus 1	pos	0						
Firmicutes	Coprococcus 2	pos	0						
Firmicutes	Coprococcus 3	pos	0						
Firmicutes	Dialister	pos	0						
Firmicutes	Dorea	pos	0						
Firmicutes	Erysipelotrichaceae UCG-003	pos	0						

Shared				SIBO neg-unique			SIBO pos-unique		
Firmicutes	Faecalibacterium	pos	0						
Firmicutes	Flavonifractor	pos	0						
Firmicutes	Fusicatenibacter	pos	0						
Firmicutes	GCA-900066575	pos	0						
Firmicutes	Gemella	pos	6						
Firmicutes	Holdemanella	pos	0						
Firmicutes	Howardella	pos	0						
Firmicutes	Lachnoclostridium	pos	6						
Firmicutes	Lachnospira	pos	0						
Firmicutes	Lachnospiraceae FCS020 group	pos	0						
Firmicutes	Lachnospiraceae ND3007 group	pos	0						
Firmicutes	Lachnospiraceae NK4A136 group	pos	0						
Firmicutes	Lachnospiraceae UCG-001	pos	6						
Firmicutes	Lachnospiraceae UCG-004	pos	6						
Firmicutes	Lachnospiraceae UCG-010	pos	6						
Firmicutes	Lactobacillus	pos	0						
Firmicutes	Megamonas	pos	0						
Firmicutes	Megasphaera	pos	6						
Firmicutes	NA	pos	0						
Firmicutes	NA	pos	6						
Firmicutes	Negativibacillus	pos	0						

Shared				SIBO neg-unique			SIBO pos-unique		
Firmicutes	Phascolarctobacterium	pos	6						
Firmicutes	Romboutsia	pos	0						
Firmicutes	Ruminiclostridium 5	pos	0						
Firmicutes	Ruminococcaceae NK4A214 group	pos	6						
Firmicutes	Ruminococcaceae UCG-002	pos	6						
Firmicutes	Ruminococcaceae UCG-003	pos	6						
Firmicutes	Ruminococcaceae UCG-010	pos	0						
Firmicutes	Ruminococcaceae UCG-014	pos	0						
Firmicutes	Ruminococcus 1	pos	6						
Firmicutes	Ruminococcus 2	pos	0						
Firmicutes	Streptococcus	pos	6						
Firmicutes	Subdoligranulum	pos	0						
Firmicutes	UBA1819	pos	0						
Firmicutes	Veillonella	pos	6						
Fusobacteria	Fusobacterium	pos	6						
NA	NA	pos	0						
Proteobacteria	Bilophila	pos	6						
Proteobacteria	Desulfovibrio	pos	0						
Proteobacteria	Escherichia-Shigella	pos	6						
Proteobacteria	Haemophilus	pos	6						
Proteobacteria	NA	pos	6						
Proteobacteria	Pseudomonas	pos	0						

Shared				SIBO neg-unique			SIBO pos-unique		
Proteobacteria	Sutterella	pos	6						
Verrucomicrobia	Akkermansia	pos	6						
Actinobacteria	Actinomyces	neg							
Actinobacteria	Senegalimassilia	neg							
Actinobacteria	Slackia	neg							
Bacteroidetes	Bacteroides	neg							
Bacteroidetes	Odoribacter	neg							
Bacteroidetes	Prevotella 9	neg							
Firmicutes	[Eubacterium] eligens group	neg							
Firmicutes	[Eubacterium] ruminantium group	neg							
Firmicutes	Catenibacterium	neg							
Firmicutes	Family XIII AD3011 group	neg							
Firmicutes	Lachnospiraceae UCG-008	neg							
Firmicutes	Oscillibacter	neg							
Firmicutes	Roseburia	neg							
Firmicutes	Ruminiclostridium 9	neg							
Firmicutes	Ruminococcaceae UCG-005	neg							
Firmicutes	Ruminococcaceae UCG-013	neg							
Proteobacteria	Parasutterella	neg							
